# Locally generated C3 regulates the clearance of *Toxoplasma gondii* by IFN-γ-primed macrophage through regulation of xenophagy

**DOI:** 10.3389/fmicb.2022.944006

**Published:** 2022-08-04

**Authors:** Bo Liu, Yan Yan, Xiaoreng Wang, Nannan Chen, Jue Wu

**Affiliations:** ^1^Department of Hematology. The No. 967 Hospital of PLA Joint Logistics Support Force, Dalian, Liaoning, China; ^2^Translational Medicine Research Center, Medical Innovation Research Division, The Chinese PLA General Hospital, Beijing, China; ^3^Laboratory of Radiation Injury Treatment, Medical Innovation Research Division, The Chinese PLA General Hospital, Beijing, China

**Keywords:** autophagy, xenophagy, macrophage, *Toxoplasma*, C3

## Abstract

Exogenous pathogen infection can induce autophagy in cells. Autophagy is essential for cell survival, development, and homeostasis. It not only regulates cell defense and stress, but also has a close relationship with innate and adaptive immunity. Complement is an important part of innate immunity, which could be activated by three approaches, including classic, alternative, and lectin pathways. All the three pathways result in the activation of C3, and generate anaphylatoxin fragments C3a and C5a, and formation of the membrane attack complex. Either C3a or C5a induces the inflammatory cytokines through binding to C3aR or C5aR, respectively. However, it is still unknown whether the complement could regulate the autophagy of intracellular microorganisms or not. In this study, we constructed a *Toxoplasma gondii* (*T. gondii*) and macrophages co-culture experimental model *using T. gondii* expressing enhanced green fluorescence protein (EGFP) fluorescence and C3^−/-^C57BL/6 J mice for that *T. gondii* invaded peritoneal macrophages in mice. Western blot, laser confocal microscopy (LCM), and transmission electron microscopy (TEM) were used to observe the changes of autophagy between the macrophages from wild-type (WT) and C3^−/−^ mice. Flow cytometry and LCM were used to investigate the effect of autophagy on the killing ability of macrophages against *T. gondii*. Here, we found that local C3 could suppress not only the canonical autophagy of macrophage, but also the xenophagy to *T. gondii*. Interestingly, the inhibition of C3 on host cell autophagy could significantly suppress the clearance of *T. gondii* by the IFN-γ-primed macrophage. Finally, we investigated the mechanism of the autophagy regulation of C3 that the effect of C3 on the macrophage-specific autophagy against *T. gondii* depends on mTOR. And, there is C3a but not C5a/C5aR involved in regulating macrophage xenophagy against *T. gondii*. Collectively, our findings suggest locally generated C3 regulates the clearance of *T. gondii* by Macrophage through the regulation of the non-canonical IFN-γ-dependent autophagy pathway, and paint a clearer picture in the regulation of autophagy by innate immune components.

## Introduction

Autophagy is a regulatory mechanism of cellular defense in response to nutrient stress (Glick et al., 2010). When cells are subjected to stresses, such as starvation and poor physiological conditions, they form autophagosomes to encapsulate aging organelles and other waste materials and fuse them with lysosomes to form autolysosomes (Lorincz and Juhasz, 2020). The autolysosomes can digest and degrade the contents into nutrients that cells can use to sustain themselves. Autophagy is an important innate immune mechanism by which the body inhibits or eliminates intracellular pathogens (Cheng et al., 2022). Recent studies have found that infection can also induce host cell autophagy (Huang et al., 2022). Cells can clear bacteria and viruses through autophagy. Autophagy does not happen in isolation, and other innate immune responses can regulate the occurrence and development of autophagy to varying degrees (Cui et al., 2019). Complement is the first line of defense against infection in the body and is an important part of innate immunity. It can be rapidly activated through the classical pathway, the alternative pathway, or the lectin pathway in the early stage of infection (Defendi et al., 2020). It has the functions of cytolysis, opsonization, inflammatory mediators, clearance of immune complexes, and immune regulation (Meuleman et al., 2022). Complement activation can produce membrane attack complexes (MACs) and key intermediates, such as complement 3a (C3a) and complement 5a (C5a) (Ostrycharz and Hukowska-Szematowicz, 2022). However, there are no relevant reports on whether complement can directly regulate the occurrence and development of autophagy.

*Toxoplasma gondii (T. gondii)* is an opportunistic parasite, and its infection is not organ-specific and is zoonotic. Toxoplasmosis is a great threat to pregnant women, fetuses, and patients with weakened immune systems (Parvin et al., 2022). It mainly affects the central nervous system (CNS) (Schluter and Barragan, 2019). *T. gondii* are targeted by a non-canonical autophagy pathway in IFN-γ-activated cells to restrict parasite growth (Selleck et al., 2015; Subauste, 2021). *T. gondii* is an important model for studying infection and immunity and is a relatively mature *in vivo* and *in vitro* infection model. Therefore, this study used *T. gondii*-infected macrophages as the research model to investigate whether complement C3 could regulate macrophage autophagy against *T. gondii* and its possible molecular mechanisms.

The theory, proposed here, describes that C3 could regulate macrophage autophagy and thus affect *T. gondii* clearance. Supported by reliable experimental data, we propose that locally generated C3 could suppress not only the canonical autophagy of macrophage, but also the xenophagy of *T. gondii*. The inhibition of C3 on host cell autophagy could significantly suppress the clearance of *T. gondii* by the IFN-γ-primed macrophage. We have made progress in understanding the regulation of autophagy by other innate immune components in the elimination of *T. gondii* in host cells. Strategies to prevent toxoplasmosis from targeting by complement blocking autophagy may represent a feasible avenue to develop novel ancillary approaches to improve the treatment of toxoplasmosis.

## Materials and methods

### Mice and parasites

The animal experiment procedure was approved by the Experimental Animal Ethics Committee of the Chinese PLA General Hospital, Beijing, China. The RH-EGFP strain of *T. gondii* used in this study was provided by Zhao-rong Lun laboratory of Sun Yat-sen University (Guangdong, China). The RH-EGFP strain of *T. gondii* was preserved in Kunming mice by passage. Kunming mice derived from Swiss mice are the most produced and used most mice in China. Kunming mice (male and female, 16–20 g, 6–8 weeks) were provided by the Laboratory Animal Center of the Third Military Medical University. Wild-type Balb/c mice (female, 16–20 g, 6–8 weeks) and C57BL/6 mice (female, 16–20 g, 6–8 weeks) are provided by the Laboratory Animal Center of Peking Union Medical College. C5aR^−/-^Balb/c mice (female, 16–20 g, 6–8 weeks) and C3^−/-^C57BL/6 mice (female, 16–20 g, 6–8 weeks) were provided by Boguo and Gui-lian Xu laboratory in the Institute of Immunology of Third Military Medical University.

### Resuscitation, passage, and cryopreservation of the RH strain of *Toxoplasma gondii*

Resuscitation: The cryopreservation tube of *T. gondii* was removed from the liquid nitrogen tank and quickly thawed in a 37°C thermostat water bath. The thawed *T. gondii* solution was aspirated into a 1-ml syringe and transferred into a 10-ml centrifuge tube. After adding 4 ml phosphate-buffered saline (PBS) (Gibco, 10,010,023, Grand Island, NY), the solution was centrifuged at 900 *g* for 15 min. Then, the supernatant was removed, and 1 ml PBS was added and gently mixed. Each Kunming mouse was intraperitoneally injected with 200 μl of this *T. gondii* solution under aseptic conditions.

Passage: At 72 h after inoculation, 8 ml of PBS was injected into the peritoneal cavity of mice with repeated pipetting and lavage, and peritoneal lavage fluid was collected in a 10-ml centrifuge tube and centrifuged at 900 *g* for 15 min. The supernatant was removed, 1 ml PBS was added and gently mixed, and each mouse was intraperitoneally injected with 200 μl of this solution under aseptic conditions.

Cryopreservation: The peritoneal lavage fluid of mice infected with *T. gondii* (same as above) was centrifuged. After adding 900 μl Dulbecco’s modified Eagle’s medium (DMEM) (Gibco, 11,965,092, Grand Island, NY), the solution was placed in a cryopreservation tube, and 100 μl dimethyl sulfoxide (DMSO) (Thermo Scientific, 85,190, Carlsbad, CA) was added and mixed well. The labeled solution was placed in a liquid nitrogen tank for long-term cryopreservation after gradient cooling.

### Establishment of a co-culture model for *Toxoplasma gondii* and macrophages

All the animal care and experimental protocols complied with the Animal Management Standards of the Ministry of Health, People’s Republic of China (documentation 55, 2001) and were approved by the local Animal Ethics Committee.

C5aR knock-out mice were obtained from the Institute of Immunology. Induction and collection of mouse peritoneal macrophages: PBS containing 3% brewer thioglycollate medium (Thermo Fisher Scientific Inc., CM0023B, Waltham, MA) was injected into the peritoneal cavity of mice. Each mouse was injected with 1 ml PBS for 3 consecutive days. Three days later, the peritoneum of mice was washed with PBS, and the lavage fluid was collected in a 10-ml sterile glass centrifuge tube and centrifuged at 220 g for 5 min. The supernatant was discarded, and an appropriate amount of DMEM containing 10% fetal bovine serum (Gibco, 10,100,147, Grand Island, NY) and 1% penicillin/streptomycin (Gibco, 10,378,016, Grand Island, NY) was added and mixed. The well-mixed cell suspension was evenly distributed into cell culture flasks. The cell culture flasks were placed in a 37°C incubator for 2 h. At this time, the macrophages were adherent, the supernatant in the cell culture flask was discarded, and the cells were washed twice with PBS. Then, 1 ml DMEM complete medium was added, and the macrophages were gently scraped off the bottom surface of the culture bottle with a cell culture scraper. Cells were harvested by centrifugation and were counted. Several 24-well plates were labeled for LCM, each well was pretreated with 20 μl complete medium, and a cover slip of 8 mm × 8 mm that had been soaked in acid, cleaned, and ultraviolet-sterilized was put in each well, where it had no room to move in the bottom of the well. In each well, 3 × 10^5^ cells were cultured overnight.

Collection of *T. gondii*: On the next morning, the peritoneal lavage fluid with *T. gondii* that had been passaged for 72 h was collected and centrifuged at 120 g for 5 min to remove peritoneal macrophages. The supernatant was collected and transferred into a new 10-ml disposable sterile plastic centrifuge tube and centrifuged at 900 *g* for 15 min. *T. gondii* were collected and counted in a cell counter.

Co-culture of *T. gondii* with macrophages: *T. gondii* and cells were put into cell culture wells at a ratio of 1:1, and the plates were centrifuged at 450 g for 30 s to sink the *T. gondii* to the bottom of the wells. The plates were placed in a 37°C cell incubator.

### Western blot

Macrophages were lysed with Radio-Immunoprecipitation Assay (RIPA) lysis buffer containing Phenylmethanesulfonyl fluoride (PMSF) (Applygen Technologies, C1055, Beijing, China), and protein was extracted. After quantified, the protein was separated based on their size by 10% Sodium Dodecyl Sulfate PolyAcrylamide Gel Electrophoresis (SDS-PAGE) (Bio-Rad Laboratories, 5,671,034, Hercules, CA) and transferred to polyvinylidene fluoride (PVDF) transfer membranes (Merck KGaA, ipfl00010, Darmstadt, Germany). The PVDF membranes were further blocked for 1 h at room temperature, and incubated overnight with primary antibody at 4°C, then incubated with secondary antibody (with HRP label) at room temperature for 1 h. The PVDF membranes were visualized by x-ray films. Primary antibodies against LC3 (Abcam, ab63817, Cambridge, England), LAMP1 (Abcam, ab208943, Cambridge, England), P62 (Abcam, ab109012, Cambridge, England), β-actin (Abcam, ab8226, Cambridge, England), and GAPDH (Abcam, ab8245, Cambridge, England) were used in this study, respectively. Intensity was measured by ImageJ software.

### Laser confocal microscopy for observing *Toxoplasma gondii* invading macrophages and co-location with autophagy-related proteins

LCM was used to observe the GFP-positive macrophages, the number of *T. gondii*, and the copolymerization rate of autophagy-related proteins with *T. gondii* proteins.

Counting the GFP-positive macrophages (%) and the number of *T. gondii* in cells: macrophages collected from peritoneal cavity of mice were digested, counted, and plated in 24-well plates at 1 × 10^6^ cells/well. The RH-EGFP strain of *T. gondii* (5 × 10^5^) was resuspended in 200 μl DMEM complete medium containing serum and both relevant antibodies. At 2 h after invasion, staining was performed. 4% paraformaldehyde solution was added for 15 min fixation at room temperature. Staining with 4′,6-diamidino-2-phenylindole (DAPI) (Beyotime Biotechnology, P0131, Shanghai, China) was performed for 5 min. The fluorescence of RH-EGFP *T. gondii* (green) was observed under LCM (Leica, DM26000, Wetzlar, Germany). Count the number of the GFP-positive macrophages (n_1_) and the number of *T. gondii* in the GFP-positive macrophages (n_2_) in 100 macrophages. The GFP-positive macrophages (%) = n1%; parasite/cell = n_2_/n_1_.

Observing the copolymerization rate of autophagy-related proteins with *T. gondii* proteins: macrophages collected from the peritoneal cavity of mice were digested, counted, and plated in 24-well plates at 1 × 10^6^ cells/well. The RH-EGFP strain of *T. gondii* (5 × 10^5^) was resuspended in 200 μl DMEM complete medium containing serum and both relevant antibodies. At 2 h after invasion, staining was performed. 4% paraformaldehyde solution was added for 15 min fixation at room temperature. Staining with 4′,6-diamidino-2-phenylindole (DAPI) (Beyotime Biotechnology, P0131, Shanghai, China) was performed for 5 min. 0.1% Triton X-100 solution was added for 10 min to make cells transparent. Cells were blocked with 5% goat serum, 1% bovine serum albumin (BSA), and 0.05% Tween20 in PBS for 1 h. The primary antibody of LC3 (Cell Signaling Technology, 3,868, Boston, MA) and LAMP-1 (Cell Signaling Technology, 3,243, Boston, MA) was incubated with the cells at 4°C overnight, followed by incubation with the secondary antibody (Alexa Fluor® 594 Conjugate, Cell Signaling Technology, 8,889, Boston, MA) at room temperature for 1 h. Staining with 4′,6-diamidino-2-phenylindole (DAPI) (Beyotime Biotechnology, P0131, Shanghai, China) was performed for 5 min. The fluorescence of RH-EGFP *T. gondii* (green) and LC3/LAMP-1 (red) was observed under LCM (Leica, DM26000, Wetzlar, Germany). Count the number of *T. gondii* that are copolymerized with LC3/LAMP-1 protein (n_3_) in 100 RH-EGFP *T. gondii.* LC3/LAMP-1 co-location (%) = n_3_%.

### Transmission electron microscopy for observing ultrastructure of *Toxoplasma gondii*

Observation of *T. gondii* in macrophages under TEM. Co-culture of *T. gondii* with macrophages for 5 h. Parasites were harvested and resuspended with 1 ml PBS. After centrifuged (5,000 × g, 5 min, 4°C), the pellets were resuspended in 2.5% glutaraldehyde and fixed overnight at 4°C. After washed with buffer, the pellets were fixed in 100 mM cacodylate for 2.5 h. After washed with distilled water, the *pellets* were dehydrated with ethanol. It was embedded in resin at 65°C for 48 h. Then, the samples were cut into ultrathin sections and l loaded onto 300-mesh copper grids (Plano GmbH, Marburg, Germany), then stained with uranyl acetate and lead citrate. The grids were viewed under TEM (Hitachi, HT7800, Tokyo, Japan).

### Flow cytometry for detecting the invasion rate and the intracellular average number of *Toxoplasma gondii*

*Toxoplasma gondii* and macrophages were co-cultured for 20 h and incubated with IFN-γ and wortmannin. Following harvest and preparation, cells were subjected to flow cytometric analysis (SONY, SH800, Tokyo, Japan). Cells were blocked with Fc receptor blocking for 15 min and then stained with anti-CD80 antibody (Abcam, ab95548, Cambridge, England) for 30 min at room temperature. The isotype controls were used to define positive cells and determine the corresponding gate. The results were analyzed using FlowJo V10 software under the same application setup.

### ELISA assays for detecting C3a

After *T. gondii* invaded C3^−/−^ or WT macrophages for 0, 3, 6, and 12 h, the medium supernatant was collected. The level of C3a was detected by Mouse complement fragment 3a (C3a) Kit (TW-REAGENG, TW10637, Shanghai, China). Samples, standard substances, and Horseradish Peroxidase (HRP)-labeled detection antibodies were successively added into the micropores precoated with mouse C3a captured antibodies, and then incubated and thoroughly washed. The substrate Tetramethylbenzidine (TMB) is used for color rendering. The depth of color was positively correlated with the level of C3a. Measure the absorbance (OD value) at 450 nm, and calculate the sample concentration.

### Statistical analysis

Statistical analysis was performed using Student’s *t*-test in GraphPad Prism 7. 95% confidence interval (CI) for the parameters in our analysis was estimated and a two-sided *p* < 0.05 (the threshold) was defined statistically significant.

## Results

### Knock-out of local complement C3 enhances the autophagy in macrophages

**Figure 1:**
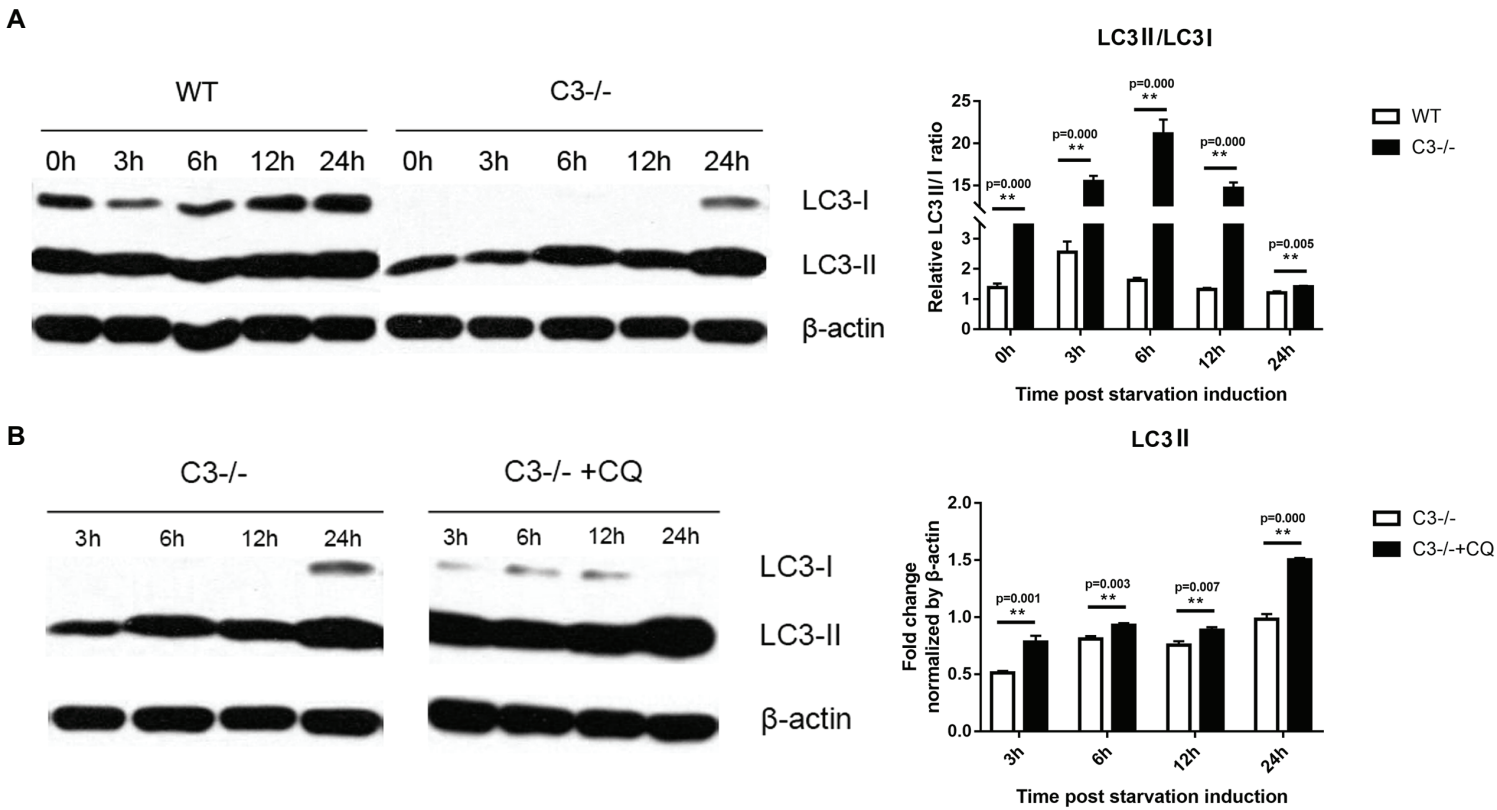
Effects of local complement C3 Knock-out on the autophagy of macrophages. **(A)** Western blot was used to detect the effect of C3 knock-out on the LC3II/LC3I ratio in macrophages at 0, 3, 6, 12, and 24 h after starvation induction. ^**^p < 0.01 indicates WT group vs. C3^−/−^ group. **(B)** Western blot was used to detect the effect of CQ on the level of LC3II in C3^−/−^ mice’ macrophages at the 3, 6, 12, and 24 h time point of starvation induction. ^**^p < 0.01 indicates C3^−/−^ group vs. C3^−/−^ + CQ group. *n* = 5 in each group. Data are the mean ± SD. WT, wild-type mice’ macrophages; C3^−/−^, macrophages from C57BL/6 J mice with C3 knock-out; C3^−/−^ + CQ, macrophages from C3^−/-^C57BL/6 J mice under starvation induction at the same time adding CQ. CQ, chloroquine. Intensity was measured by ImageJ software.

Microtubule-associated protein light chain 3 (LC3) is the first confirmed protein specifically involved in the formation of autophagosomes and is now widely used to monitor autophagy (Mizushima and Yoshimori, 2007). The amount of LC3 type II (LC3II) reflects the number of autophagosomes and autophagy-related structures. Through nutritional starvation (deprivation of serum from cell culture medium), the number of autophagosomes is increased and correspondingly, LC3II is also increased (Yoshii and Mizushima, 2017). LC3 exists in the cytosol as type I (LC3I) under normal conditions, and is recruited to autophagosomes and subsequently transformed into LC3II under autophagy-activated conditions (Mizushima, 2004). The ratio between LC3II and LC3I derived from LC3II resulted in a reliable index of autophagy formation, and detecting LC3II/LC3I ratio by immunoblotting analysis is used mostly to monitor autophagic activity (Karim et al., 2007; Jiang and Mizushima, 2015; Lu et al., 2018). However, antibodies tend to have a greater affinity for LC3II, and sometimes, LC3I could appear very faint depending on the antibody and cell types (Yoshii and Mizushima, 2017). In this case, it is recommended to compare the amount of LC3-II with one of the housekeeping proteins (Jiang and Mizushima, 2015). Macrophages from C3^−/−^ and wild-type (WT) mice at 3, 6, 12, and 24 h after starvation induction were collected, and Western blots were run to compare the expression of LC3 protein between the two groups of cells. The results showed that the LC3II/LC3I ratios in macrophages from C3^−/−^ mice were significantly higher than that in the WT group (Figure 1A). These results indicated that C3 could significantly inhibit classical autophagy in macrophages.

Although autophagy and related processes are dynamic, they can still be divided into several discrete steps: induction, formation of autophagosome, formation of autophagolysosome, transmission, and degradation of autophagosome. Chloroquine (CQ) is an inhibitor of the fusion of autophagosomes and lysosomes (Xu et al., 2018). To determine the inhibition of C3 on autophagy is at which discrete stage of autophagy flow, CQ was added at different time points to observe its effect on the LC3II of macrophages from C3^−/−^ mice. The results showed that CQ increased the level of LC3II in macrophages from C3^−/−^ mice at each time point compared with the C3^−/−^ group (non-CQ group) (Figure 1B). The above results indicated that in macrophages, the inhibition of autophagy by C3 did not inhibit the fusion of autophagosomes and lysosomes in autophagy flow. The above results indicate that the inhibition of autophagy by C3 knock-out in macrophages does not act on the fusion of autophagosome and lysosome in the autophagy flow, but may affect the downstream of the fusion of autophagosome and lysosome in the autophagy flow in autophagy flow.

### Knock-out of local complement C3 promotes specific autophagy against *Toxoplasma gondii* in macrophages

**Figure 2:**
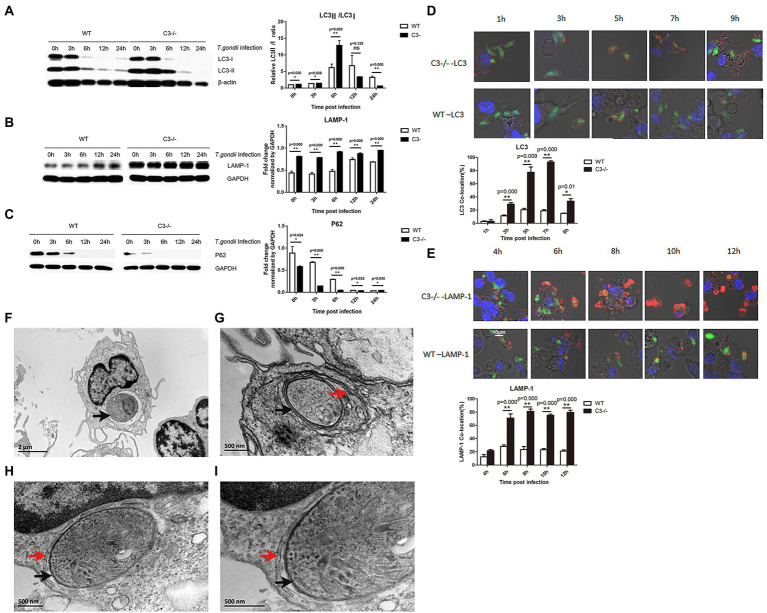
Knock-out of local complement C3 promotes the specific autophagic activity against *T. gondii* in macrophages. **(A-C)** Western blot was used to detect the effect of *T. gondii* infection on LC3, LAMP-1, and p62 protein expression in macrophages from C3^−/−^ and WT mice at 0, 3, 6, 12, and 24 h time points. Intensity was measured by ImageJ software. **(D)** Copolymerization ratio of LC3 with intracellular *T. gondii* proteins in macrophages from C3^−/−^ and WT mice at 1, 3, 5, 7, and 9  h time points. **(E)** Copolymerization ratio of LAMP-1 with intracellular *T. gondii* proteins in macrophages from C3^−/−^ and WT mice at 4, 6, 8, 10, and 12 h time points. The images were obtained by a confocal microscope. LC3 and LAMP-1 appear red fluorescence, and intracellular *T. gondii* proteins appear green fluorescence. Count the number of *T. gondi*i that are copolymerized with LC3/LAMP-1 protein (n) in 100 RH-EGFP *T. gondii.* LC3/LAMP-1 co-location (%) = n%. ^*^p < 0.05, ^**^p < 0.01 indicates WT group vs. C3^−/−^ group. *n* = 5 in each group. Data are the mean ± SD. WT, wild-type mice’ macrophages; C3^−/−^, macrophages from C57BL/6 J mice with C3 knock-out. Time points,the time for macrophages incubated with the RH-EGFP *T. gondii*. Scale bars, 10 μm. **(F-I)** Ultrastructure of *T. gondii* in C3^−/−^ macrophages by TEM. **(F)** An image from a longitudinal slice of macrophages from WT mice invaded by *T. gondii.* G-I. An image from a transverse slice of macrophages from C3^−/−^ mice invaded by *T. gondii.* **(I)** is magnified from **(H)**. The black arrows point to the parasitophorous vacuole membrane. The red arrows point to the double-membrane vesicle structure. Scale bars, 2  μm and 500 nm in magnified.

In addition to maintaining homeostasis through the canonical autophagy, cells can also remove invading pathogens by up-regulating selective autophagy (xenophagy). “xenophagy” is a specialized autophagy system in mammals, for the clearance of invading pathogens, whereas bacteria secrete proteins to defend and escape from the host xenophagy (Levine, 2005; Kwon and Song, 2018; Besteiro, 2019). We investigated whether C3 knock-out in macrophages affected xenophagy of *T. gondii*. Peritoneal macrophages were incubated with *T. gondii* using enhanced green fluorescence protein (EGFP)-labeled RH tachyzoites (RH-EGFP), and the LC3II/LC3I ratios in macrophages from C3^−/−^ and WT mice at different time points (0, 3, 6, 12, and 24 h) were analyzed by Western blot. The results showed that the LC3II/LC3I ratios of macrophages from C3^−/−^ mice were significantly higher than that of the control group 6 h before the incubation of peritoneal macrophages (Figure 2A).

Although the specific mechanism of selective autophagy is still not clear, ubiquitination of target pathogens plays a key role. Ubiquitinated proteins, such as p62 (You et al., 2019), can recognize and ubiquitinate invading pathogens. LC3II recognizes these ubiquitinated pathogens and encapsulates them in autophagosomes. Our results showed that C3 knock-out in macrophages enhanced xenophagy of *T. gondii* and autophagosome formation (Figure 2A). As autophagy progresses, these ubiquitinated proteins are gradually degraded. Therefore, the detection of the dynamic expression levels of ubiquitinated proteins is considered an important marker of autophagosome formation (Chu et al., 2021). As shown in Figure 2C, the expression of ubiquitinated p62 in macrophages from C3^−/−^ and WT mice showed a gradual decreasing trend with time, and macrophages from C3^−/−^ mice had significantly lower p62 at all detection time points than control cells.

Lysosome-associated membrane protein 1 (LAMP-1) is an important marker protein for the fusion of autophagosomes and lysosomes. LAMP-1 can protect the lysosomal membrane against the action of hydrolytic enzymes (Eskelinen, 2006). When we detected LAMP-1 in macrophages from C3^−/−^ and WT mice (Figure 2B), its expression in macrophages from C3^−/−^ mice at all detection time points was significantly higher than that in the control group, which further confirmed that complement C3 could significantly inhibit the selective autophagy against *T. gondii* in macrophages.

Since Western blot results can only confirm the differences in the expression levels of autophagy-related proteins in cells but cannot explain the specific mechanism by which cells target the invading *T. gondii* for autophagy, laser confocal microscopy (LCM) was used to observe the copolymerization rate of autophagy-related proteins with *T. gondii* proteins to directly view the autophagy against *T. gondii* in macrophages. The LCM results showed (Figures 2D,E) that as the invasion time of *T. gondii* was prolonged, the copolymerization strength of LC3 and LAMP-1 with *T. gondii* proteins was significantly enhanced, and the copolymerization in macrophages from C3^−/−^ mice at each time point was significantly stronger than that in control macrophages. Specifically, the copolymerization rate of LC3 and *T. gondii* proteins in macrophages from C3^−/−^ mice was significantly higher than that in the control group at each time. The copolymerization rate of LAMP-1 and *T. gondii* in macrophages from C3^−/−^ mice was also significantly higher than that in the control group at each time.

Transmission electron microscopy (TEM) is the gold standard for the detection of autophagy. Xenophagy of macrophages to *T. gondii* is a multistep process by which the parasitophorous vacuole membrane (PVM) is sequestered in a double-membrane vesicle termed the autophagosome and delivered to the lysosome for degradation (Besteiro, 2012). Under TEM, “a typical double-membrane vesicle structure” indicates xenophagy against *T. gondii* in macrophages. To further confirm the occurrence of autophagy against *T. gondii* in the cells after *T. gondii* invaded macrophages, TEM was used to observe the ultrastructure of macrophages from WT (Figure 2F) or C3^−/−^ mice (Figures 2G–I) after 5 h of *T. gondii* infection. As shown in Figure 2F, there is the PVM rather than a double-membrane vesicle structure in macrophages from WT. However, *T. gondii* in macrophages from C3^−/−^ mice had a typical double-membrane vesicle structure in addition to the cell membrane and the PVM (Figures 2G–I). These results indicate that the formation of autophagosome is more in macrophages from WT compared with macrophages from C3^−/−^ mice. It confirms that knock-out of C3 could enhance specific autophagy against *T. gondii* in macrophages furtherly.

### Macrophage autophagy induced by complement C3 knock-out prompts IFN-γ to remove *Toxoplasma gondii*

**Figure 3:**
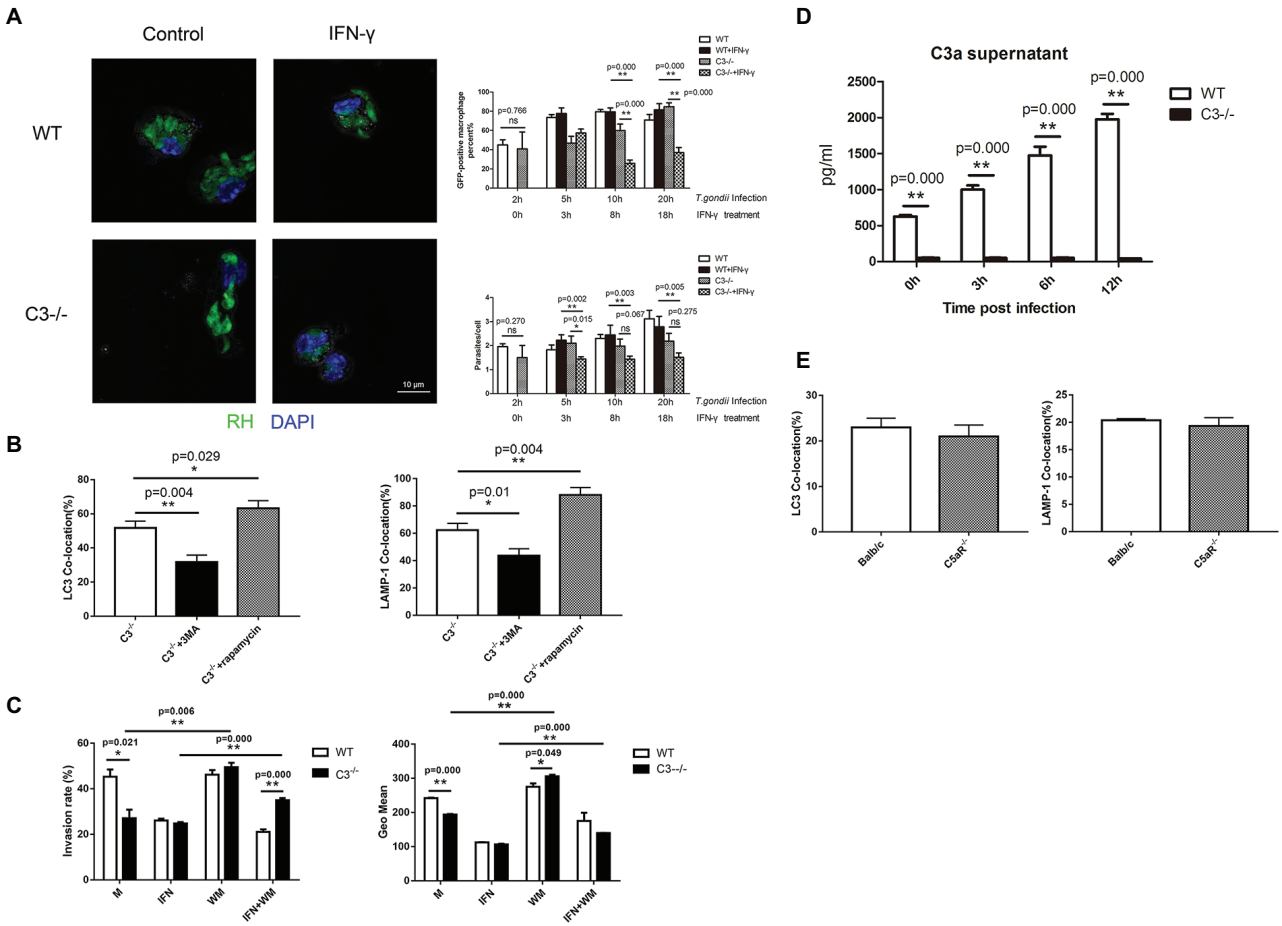
Macrophage autophagy induced by complement C3 Knock-out helps IFN-γ remove *T. gondii*. **(A)** Laser confocal microscopy is used to observe the killing of *T. gondii* by IFN-γ-pretreated macrophages from WT and C3^−/−^ mice. RH-EGFP *T. gondii* appears green fluorescence and the macrophage nucleus is stained with DAPI (blue fluorescence). Count the number of the GFP-positive macrophages (n_1_) and the number of *T. gondii* in the GFP-positive macrophages (n_2_) in 100 macrophages. The GFP-positive macrophages (%) = n_1_%; parasite/cell = n_2_/n_1_. *T. gondii* was co-cultured with macrophages from C3^−/−^ and WT mice for 2, 5, 10, and 20 h respectively, in different experimental groups. IFN-γ was added after 2 h of the co-culture of T. gondii and macrophages on each group. The horizontal axis represents the the time for the treatment. Scale bars, 10 μm. WT, wild-type mice’ macrophages; C3^−/−^, macrophages from C57BL/6 J mice with C3 knock-out; WT + IFN-γ, wild-type mice’ IFN-γ-activated macrophages. C3^−/−^ + IFN-γ, IFN-γ-activated macrophages from C57BL/6 J mice with C3 knock-out. **(B)** Laser confocal microscopy is used to observe the co-location of LC3/LAMP-1 and *T. gondii* proteins in macrophages intervened with rapamycin or 3-MA. C3^−/−^, macrophages from C57BL/6 J mice with C3 knock-out; C3^−/−^ + rapamycin, macrophages from C57BL/6 J mice with C3 knock-out that cultured with rapamycin for 5 h; C3^−/−^ + 3-MA, macrophages from C57BL/6 J mice with C3 knock-out that cultured with 3-MA for 5 h. **(C)** Flow cytometry was used to detect the invasion rate and the intracellular average number of *T. gondii.* Macrophages were stained with anti-CD80 antibody (Ex: 496 nm, Em: 670 nm). M, control group of macrophages; IFN, IFN-γ-activated macrophages; WM, macrophages treated with and wortmannin; IFN + WM, IFN-γ-activated macrophages treated with and wortmannin. **(D)** Detection of the C3a level in the culture supernatant by ELISA at 0, 3, 6, and 12 h after C3−/−and WT macrophages invaded by *T. gondii.* WT, wild-type mice’ macrophages; C3^−/−^, macrophages from C57BL/6 J mice with C3 knock-out. **(E)** The copolymerization rates between intracellular *T. gondii* proteins and LC3 and LAMP-1 in macrophages from C5aR^−/−^ and WT mice (Balb/c). Balb/c, macrophages from Balb/c mice; C5aR^−/−^, macrophages from C5aR knock-out Balb/c mice. Ns: no significance, *p* > 0.05; ^*^p < 0.05, ^**^p < 0.01. *n* = 5 in each group. Data are the mean ± SD.

Studies on *T. gondii* have shown that autophagosomes cannot directly kill *T. gondii* in parasitophorous vacuoles, but autophagy and autophagy-related proteins can promote the killing effect of the non-canonical IFN-γ-dependent autophagy pathway against *T. gondii* (Sasai et al., 2018; Zhu et al., 2019; Subauste, 2021). Next, we tried to research the effect of C3 on killing *T. gondii* by IFN-γ-activated macrophages. *In vivo* experiments on mice (Figure 3A) showed that when *T. gondii* was incubated for 2 h with peritoneal macrophages that were pretreated with IFN-γ for 18 h, the average number of *T. gondii* in each single cell in macrophages from C3^−/−^ mice was close to that in the macrophages from WT mice, but at 20 h coincubation, the invasion rate and the average number of *T. gondii* in each cell in macrophages from C3^−/−^ mice were significantly lower than those in the control group (*p* < 0.05). At the same time, *in vitro* experiments also had been taken. After *T. gondii* was incubated with macrophages for 20 h, flow cytometry was used to observe the effect of wortmannin (s a selective phosphatidylinositol 3-kinase (PI3K) inhibitor, inhibits the formation of autophagosomes), an autophagy inhibitor, on the killing of *T. gondii* by IFN-γ-pre-activated macrophages from C3^−/−^ mice. As shown in Figure 3C, consistent with the results of LCM, both the invasion rate and the intracellular average number of *T. gondii* indicated that C3 knock-out indeed significantly enhanced the killing ability of IFN-γ-activated macrophages from C3^−/−^ mice. Interestingly, the autophagy inhibitor wortmannin significantly inhibited the killing effect of IFN-γ-activated macrophages from C3^−/−^ mice on intracellular *T. gondii*, suggesting that C3 knock-out can enhance the inhibition of IFN-γ on the invasion and proliferation of *T. gondii*.

The question was, whether the effect of C3 knock-out on promoting IFN-γ-activated macrophages to kill *T. gondii* is through enhancing autophagy? It is known classic autophagy is mediated by the mammalian target of rapamycin (mTOR)-upstream of the autophagy signaling pathway (Wang and Zhang, 2019). Upstream of mTOR the survival PI3K modulates mTOR activity (Heras-Sandoval et al., 2014). Rapamycin can induce autophagy by inhibiting mTOR and is recognized as an autophagy inducer (Gao et al., 2020). 3-Methyladenine (3-MA) is a selective phosphatidylinositol 3-kinase (PI3K) inhibitor, and is a widely used classic autophagy inhibitor (Wu et al., 2010). Therefore, we explored whether the effect of C3 on macrophage-specific autophagy against *T. gondii* depends on mTOR by observing the effects of rapamycin and 3-MA on the autophagy against *T. gondii* in macrophages from C3^−/−^ mice. LCM was used to observe the copolymerization rates of LC3 and LAMP-1 with *T. gondii* cultured with rapamycin or 3-MA for 5 h in macrophages from C3−/− mice. The results showed that 3-MA significantly decreased the percentage of macrophage autophagy against *T. gondii*, while rapamycin significantly increased the percentage of macrophage autophagy against *T. gondii* (Figure 3B). These results indicate that the mTOR pathway might play an important role in autophagy of macrophage from C3−/− mice against *T. gondii*.

Complement is activated in macrophages invaded by *T. gondii*. The central step of complement activation is the conversion of C3 to C3a, which is responsible for clearing pathogens (Wu et al., 2022). Does the local activation of C3 regulate macrophage xenophagy through these intermediate products? To answer this question, we conducted the following experiment. At 0, 3, 6, and 12 h after *T. gondii* (RH-EGFP) invasion of macrophages from C3^−/−^ and WT mice, the culture supernatant was collected, and the levels of complement C3a were detected by enzyme-linked immunosorbent assay (ELISA). As shown in Figure 3D, there is no C3a in the cultural supernatant of macrophages from C3^−/−^ mice collected at any time point. In addition, the concentration of C3a in the culture supernatant of macrophage from WT mice was increased with the invasion time. These results showed that the local activation of C3 regulates macrophage xenophagy to *T. gondii* through C3a. After complement activation, C3a and C5a are generated all (Sunyer et al., 2003). Does the local activation of complement also regulate macrophage xenophagy through C5a? Meanwhile, we investigated whether C5a/C5aR signaling is involved in the regulation of autophagy in macrophages using C5aR knock-out mice. The copolymerization of LC3 and LAMP-1 with *T. gondii* was detected at 5 and 8 h after *T. gondii* invaded macrophages, and the results showed that there were no significant differences in the copolymerization rates of intracellular *T. gondii* proteins and LC3/LAMP-1 between macrophages from C5aR^−/−^ and WT mice (Figure 3E). This result indicated that the C5a/C5aR signaling pathway does not involve in macrophage xenophagy against *T. gondii*.

## Discussion

In this study, *T. gondii* expressing EGFP and C3^−/−^ C57BL/6 J mice were used to construct a *Toxoplasma gondii* (*T. gondii*) and macrophages co-culture experimental model for autophagy in mouse peritoneal macrophages invaded by *T. gondii*. The differences in the autophagy between the C3^−/−^ group and the control group were analyzed by Western blot, LCM, and TEM, and the effect of host cell autophagy on the killing of macrophages on *T. gondii* was observed using flow cytometry and LCM. The results showed that local complement C3 not only inhibited classical autophagy in macrophages but also inhibited the macrophage autophagy against *T. gondii*. In addition, inhibition of autophagy in host cells by complement C3 inhibited the clearance effect of IFN-γ on *T. gondii*. This finding enriches our understanding of the relationship between autophagy and innate immunity and is will be helpful for understanding the pathogenesis of autophagy-related diseases.

Scholars have made considerable progress in understanding the interactions between autophagy and the immune response. Complement is an important part of the innate immune system, and their defects, dysfunction, and abnormal activation are involved in the occurrence and development of a variety of diseases (Freiwald and Afzali, 2021). The complement system is a highly complex biological reaction system that is activated by the classical pathway, alternative pathway, or lectin pathway (Skinner et al., 2021). During the activation process, multiple active fragments are produced, which can mediate a variety of biological effects. C3 is the core molecule of the three activation pathways. To answer whether complement C3 affects autophagy, this study established an *in vitro* experimental model for the invasion by *T. gondii* of macrophages and repeatedly explored the time course of the macrophage autophagy induced by *T. gondii*, thereby shedding light on the effect of complement C3 on autophagy *in vitro*. Western blot, LCM, TEM, and the addition of typical autophagy inducers and inhibitors, which are widely accepted methods in the field of autophagy, were used to comprehensively and rigorously demonstrate the macrophage autophagy induced by the invasion of *T. gondii* and the effect of C3 knock-out on this autophagy against *T. gondii.* For the first time, our results show that complement C3 knock-out significantly enhanced and accelerated the autophagic activity of macrophages, specifically the autophagy against *T. gondii*. Under IFN-γ stimulation, the C3 knock-out induced enhancement of autophagy in macrophages inhibited the proliferation and development of *T. gondii* tachyzoites. This finding confirms the strong relationships between innate immunity and autophagy.

Both complement and Toll-like receptors belong to the first line of defense of the innate immune system against infection. Toll-like receptors play an important role in the regulation of autophagy (Lee et al., 2021). This study showed that complement negatively regulated autophagy, which seems contradictory. In the body, complement is mainly secreted by liver cells and dispersed in serum to play their roles in the immune response (Hu et al., 2021), and the number of complement secreted by macrophages is relatively small compared to that by liver cells. Our experiments were carried out in the context of partial complement knock-out in macrophages, so the true roles of complement may be different from their previously recognized roles.

After C3 is activated in cells, it can be lysed under the action of C3 convertase to produce C3a, C3b, and other fragments, but the C3a fragment has the highest activity (Dobo et al., 2016). Is C3a the effective fragment of C3 that regulates autophagy? The molecular mechanism of the autophagy process is quite complicated and involves the regulation of the expression levels of more than ten autophagy-related proteins. Could C3 affect autophagy by regulating the expression of certain key autophagy-related proteins? Our experiments showed that *T. gondii* infection could significantly promote complement activation in macrophages and generate active fragment C3a. There are three pathways of complement activation, i.e., the classical pathway, lectin pathway, and alternative pathway, but it is not clear which pathway mediates the activation of macrophage complement induced by *T. gondii* infection, a matter that needs to be clarified by further experiments. No matter which pathway is involved in the activation of macrophage local complement, complement activation can not only form MACs but also produce a series of intermediate products, such as C3a, C5a, and C3b (Vignesh et al., 2017). Among them, the anaphylatoxins C3a and C5a are important active fragments that function as inflammatory mediators (Gal et al., 2009). The negative regulation of autophagy by C3 observed here might be due to the action of these active fragments. We conducted LCM on the available C5aR^−/−^ mice in our laboratory, and the statistical analysis of the LCM data indicated that C5a/C5aR did not affect the macrophage autophagy against *T. gondii*. However, to further confirm the above results, it is necessary to antagonize C5aR *in vitro* to find out whether C5a/C5aR signaling affects macrophage autophagy.

## Conclusion

We firstly reported that the complement C3 could greatly inhibit both the canonical autophagy and xenophagy of macrophage, and suppress the clearance of *T. gondii* by IFN-γ-primed macrophage. Although the actual regulatory mechanism of autophagy by complement is still unknown, C5a/C5aR signaling is not involved in this process. These data increase our understanding of the crossing-talking between the autophagy and innate immune response, and suggest a novel role of local complement to promote the growth of intracellular pathogen through negative regulation of autophagy.

## Data availability statement

The raw data supporting the conclusions of this article will be made available by the authors, without undue reservation.

## Author contributions

JW and NC contributed to the conception and design of the study. YY performed the statistical analysis. BL wrote the first draft of the manuscript. XW wrote sections of the manuscript. All authors contributed to the article and approved the submitted version.

## Funding

This work was supported by grants from the Hygiene and Health Development Scientific Research Fostering Plan of Haidian District Beijing (HP2021-25-80101 and HP2021-25-80102).

## Conflict of interest

The authors declare that the research was conducted in the absence of any commercial or financial relationships that could be construed as a potential conflict of interest.

## Publisher’s note

All claims expressed in this article are solely those of the authors and do not necessarily represent those of their affiliated organizations, or those of the publisher, the editors and the reviewers. Any product that may be evaluated in this article, or claim that may be made by its manufacturer, is not guaranteed or endorsed by the publisher.

## Supplementary material

The Supplementary material for this article can be found online at: https://www.frontiersin.org/articles/10.3389/fmicb. 2022.944006/full#supplementary-material
