# Supplementary figures and images for: Locally generated C3 regulates the clearance of Toxoplasma gondii by IFN-γ-primed macrophage through regulation of xenophagy
Source: Front Microbiol. 2022 Aug 4;13:944006. doi: 10.3389/fmicb.2022.944006 (PMC9386420; doi:10.3389/fmicb.2022.944006)

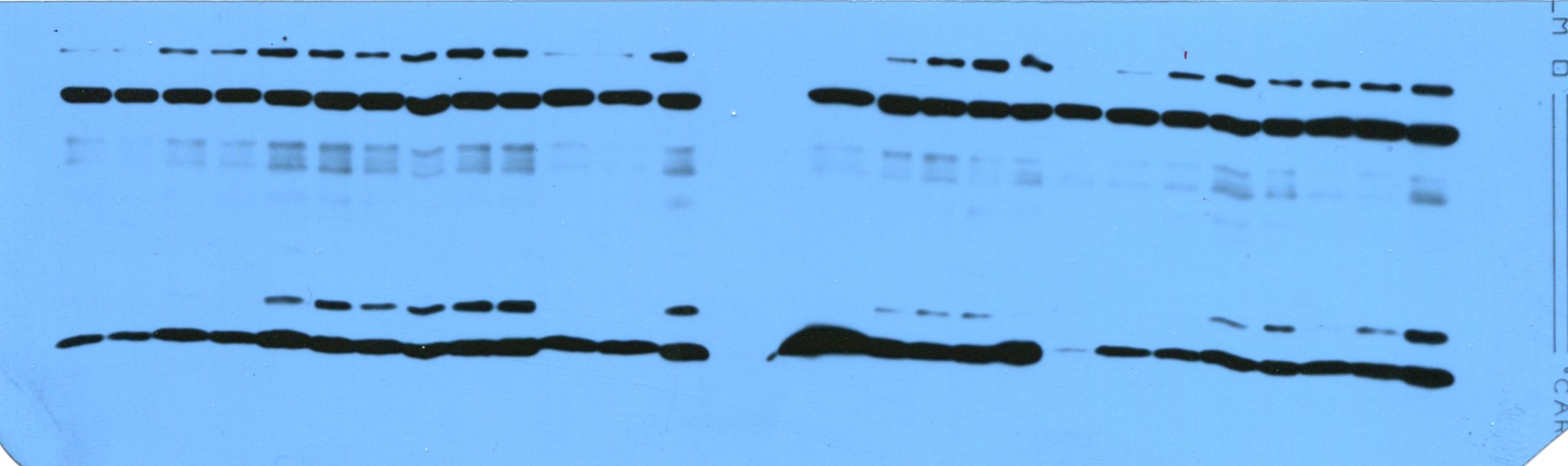

Supplement: Supplementary file 1 [file Data_Sheet_1.ZIP › Fig1/Fig1A+B (full scans of the entire original gels).jpg]

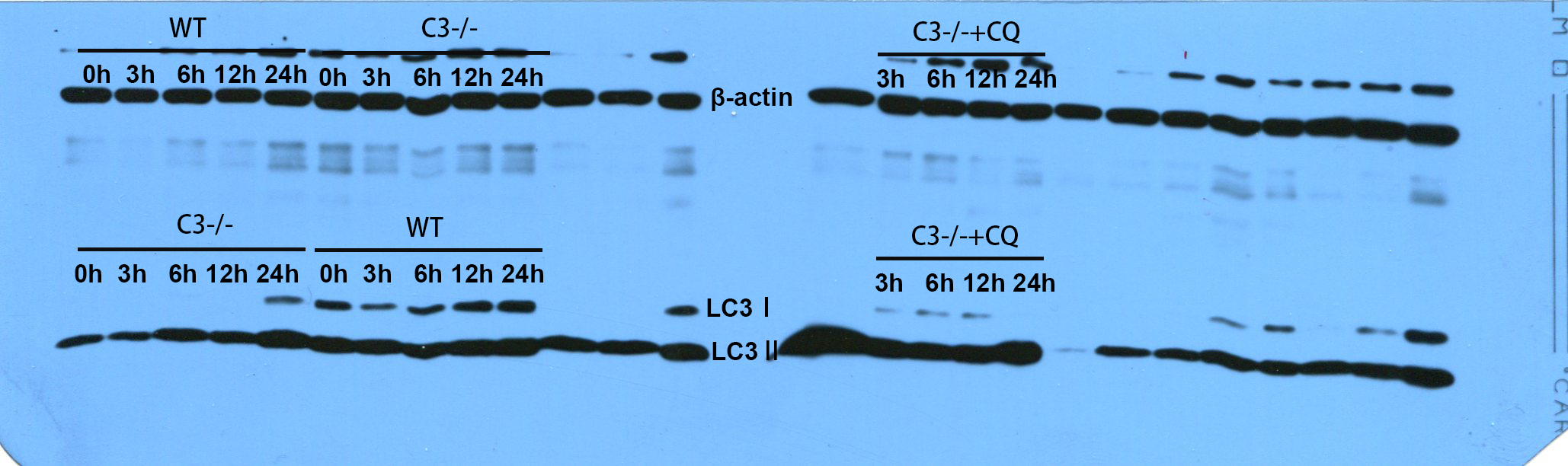

Supplement: Supplementary file 1 [file Data_Sheet_1.ZIP › Fig1/Fig1A+B (with mark).jpg]

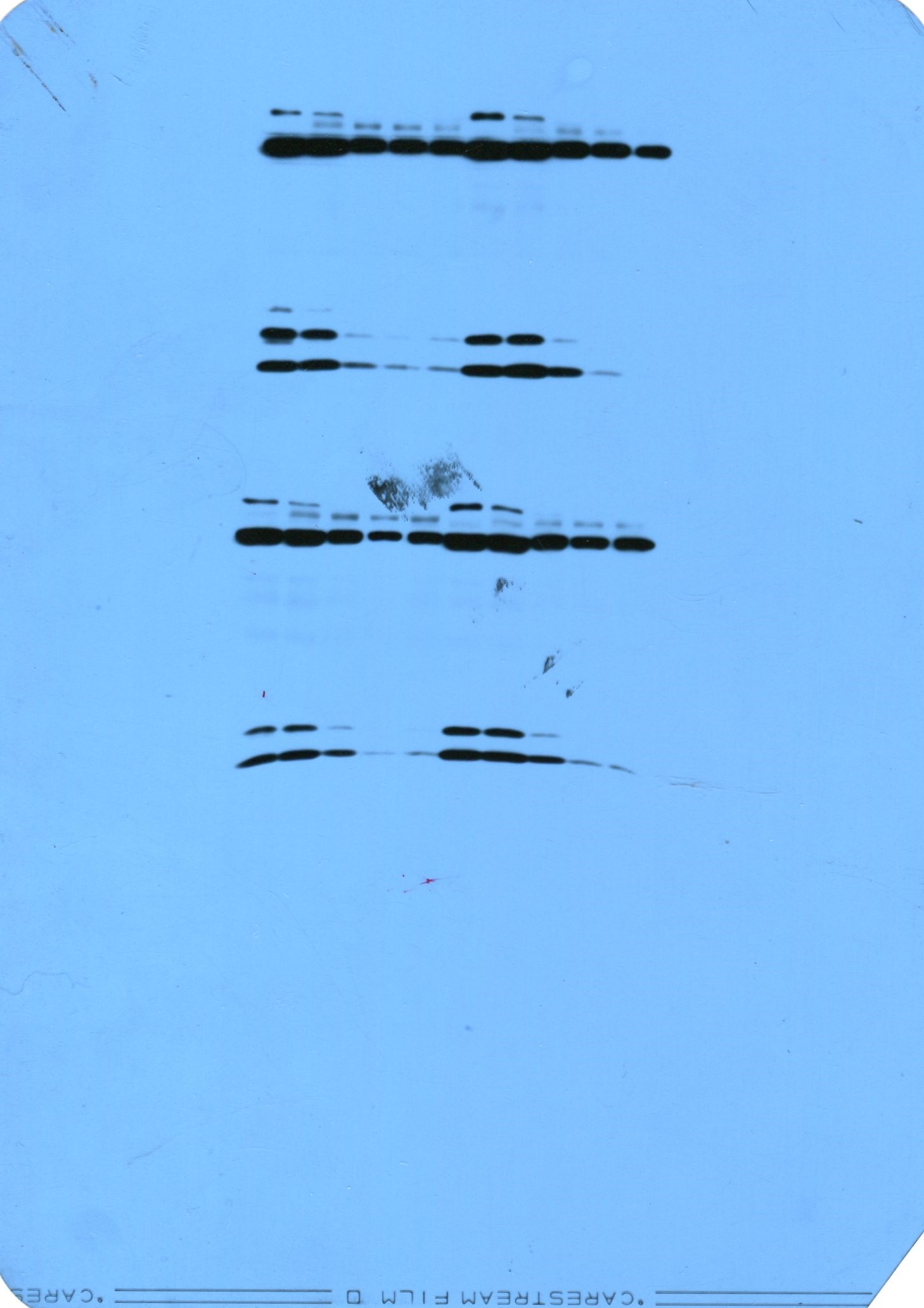

Supplement: Supplementary file 2 [file Data_Sheet_2.zip › Fig2/Fig2A-C/Fig2A (full scans of the entire original gels).jpg]

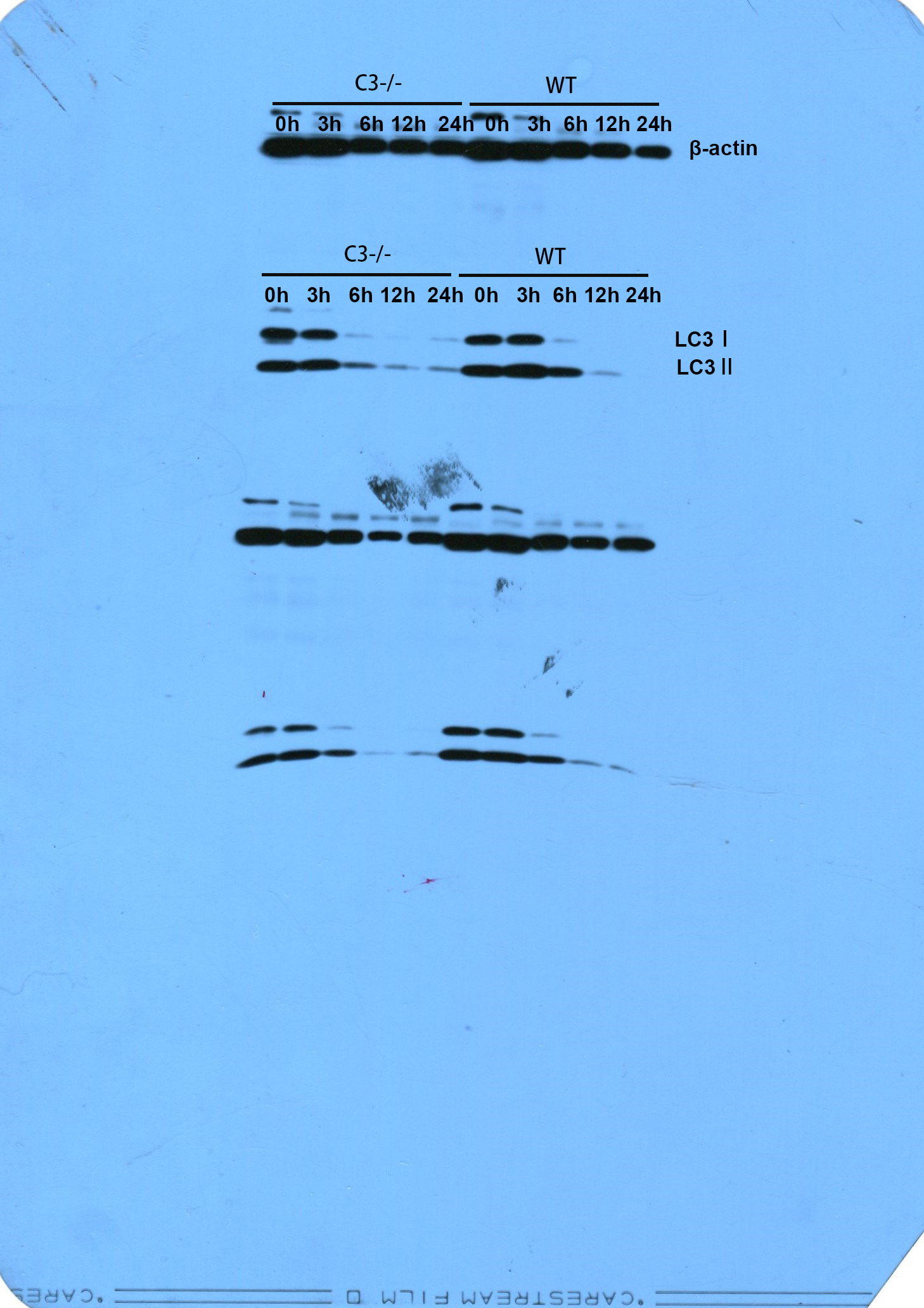

Supplement: Supplementary file 2 [file Data_Sheet_2.zip › Fig2/Fig2A-C/Fig2A (with mark).jpg]

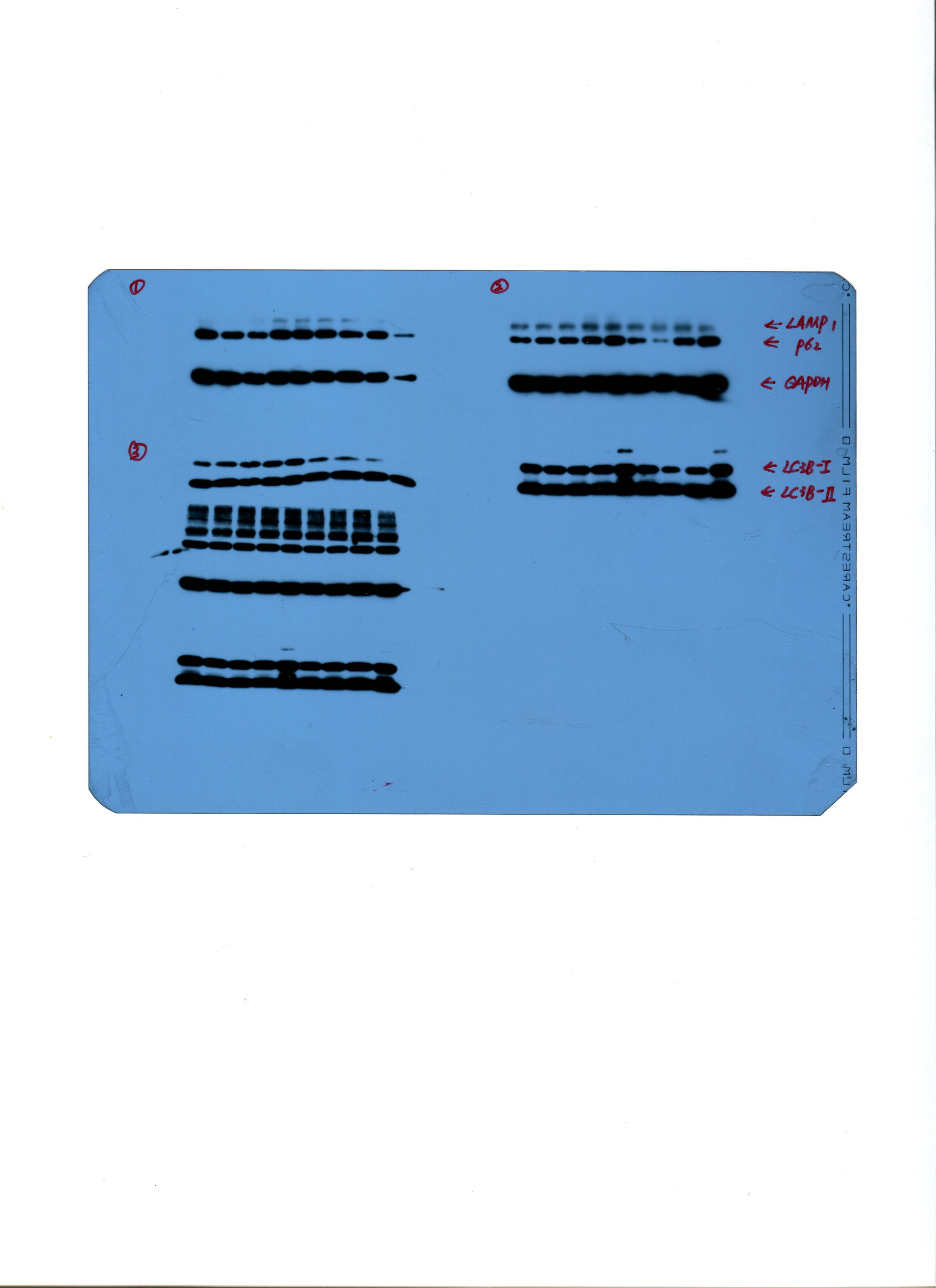

Supplement: Supplementary file 2 [file Data_Sheet_2.zip › Fig2/Fig2A-C/Fig2B (full scans of the entire original gels).jpg]

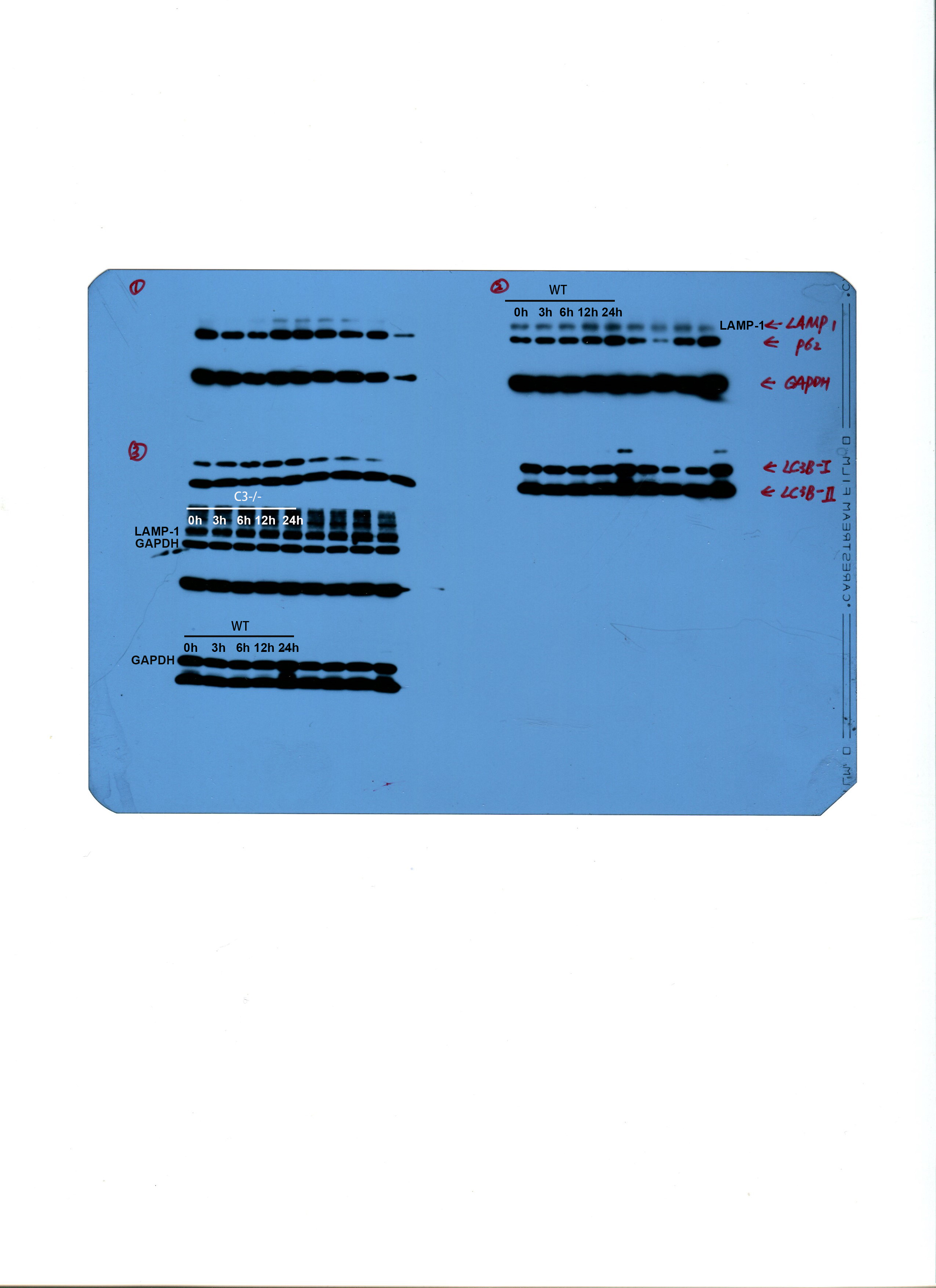

Supplement: Supplementary file 2 [file Data_Sheet_2.zip › Fig2/Fig2A-C/Fig2B (with mark).jpg]

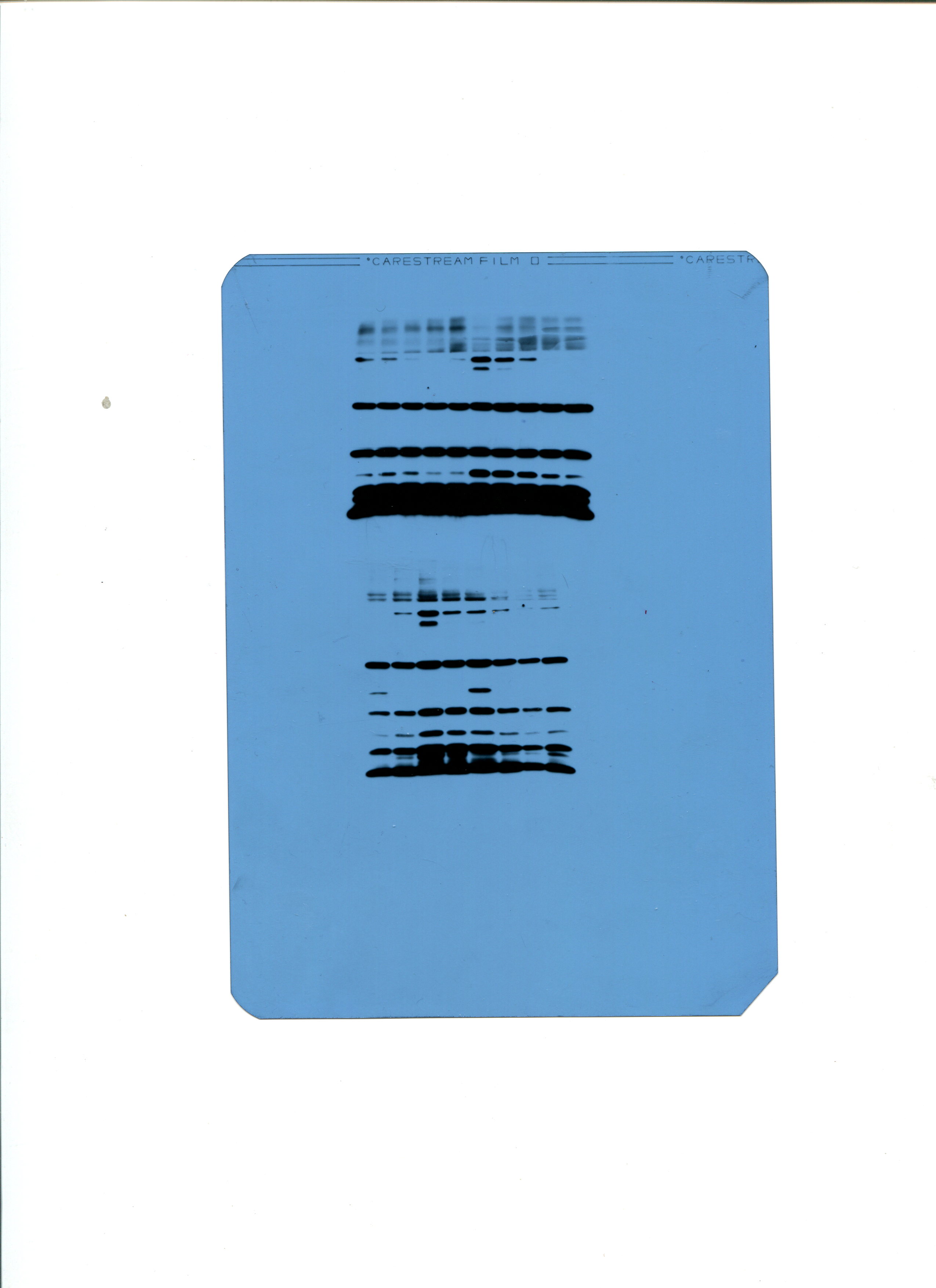

Supplement: Supplementary file 2 [file Data_Sheet_2.zip › Fig2/Fig2A-C/Fig2C (full scans of the entire original gels).jpg]

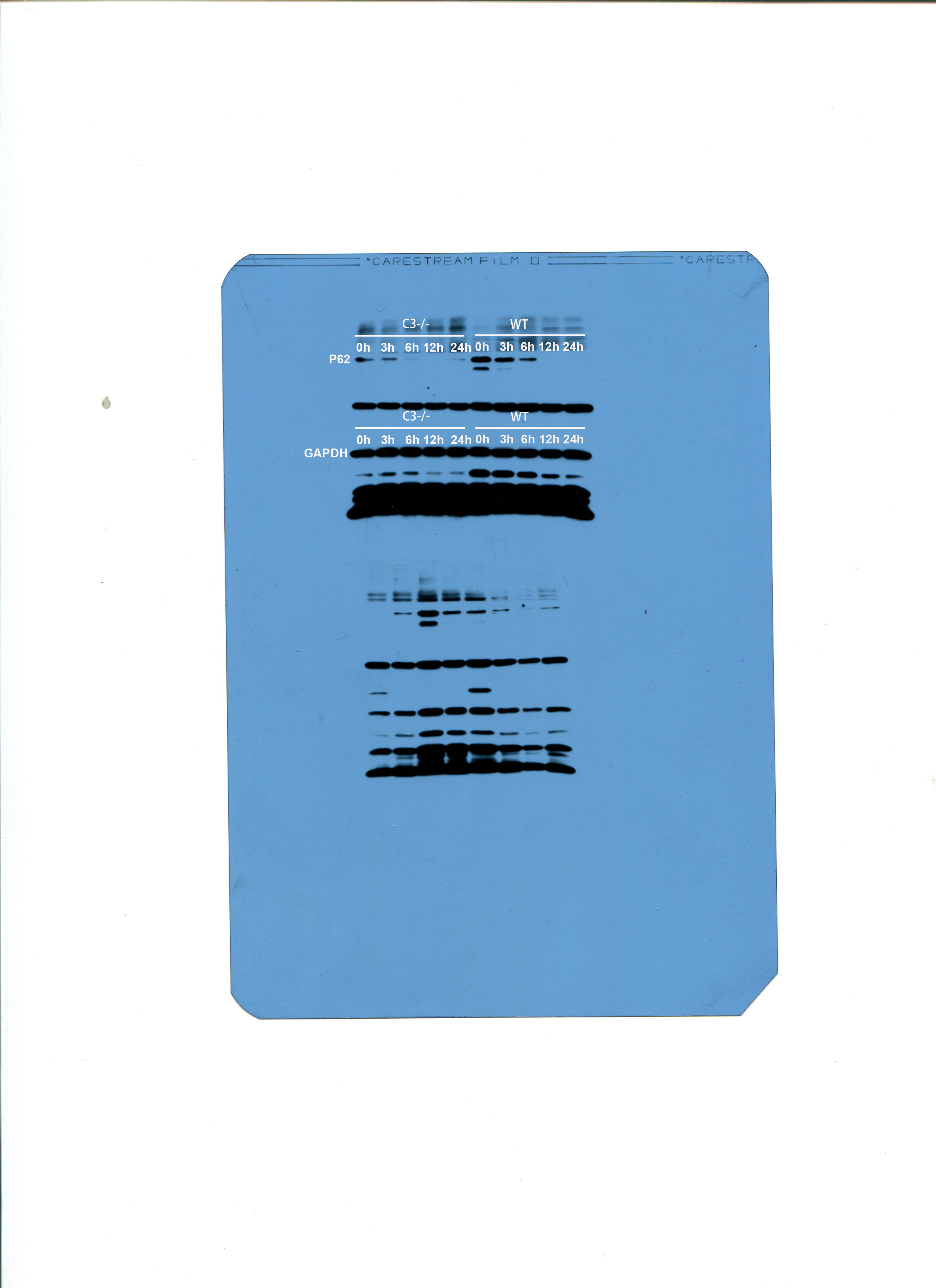

Supplement: Supplementary file 2 [file Data_Sheet_2.zip › Fig2/Fig2A-C/Fig2C (with mark).jpg]

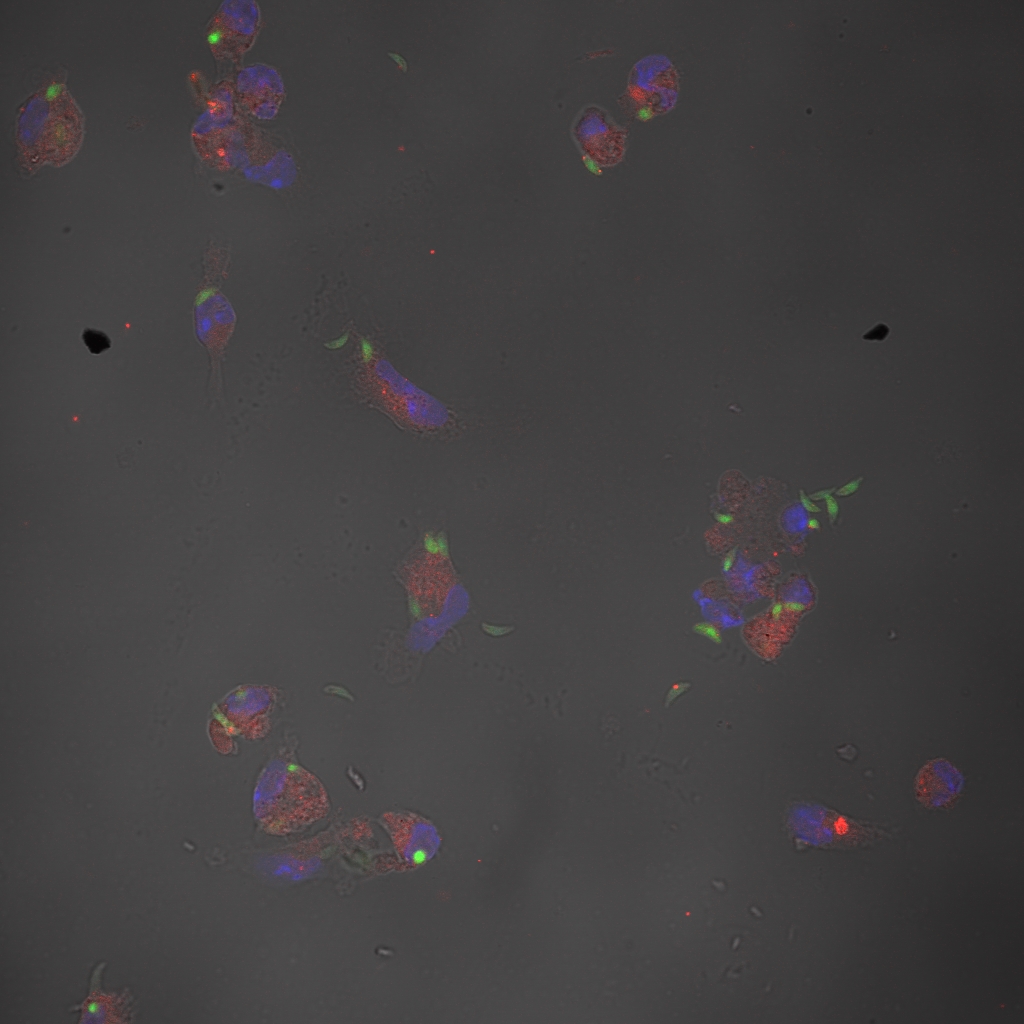

Supplement: Supplementary file 2 [file Data_Sheet_2.zip › Fig2/Fig2D/C3-LC3-1h/C3-LC3-1h.jpg]

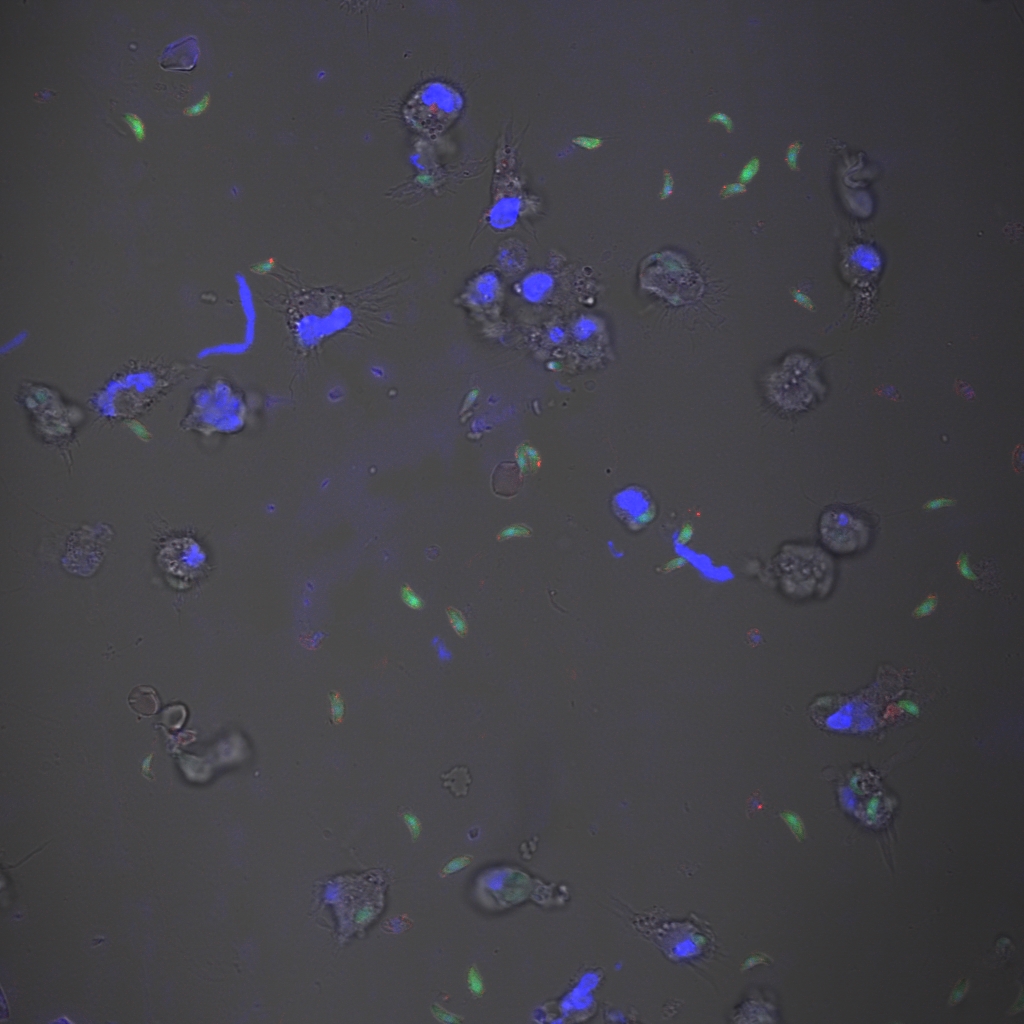

Supplement: Supplementary file 2 [file Data_Sheet_2.zip › Fig2/Fig2D/C3-LC3-3h/C3-LC3-3h.jpg]

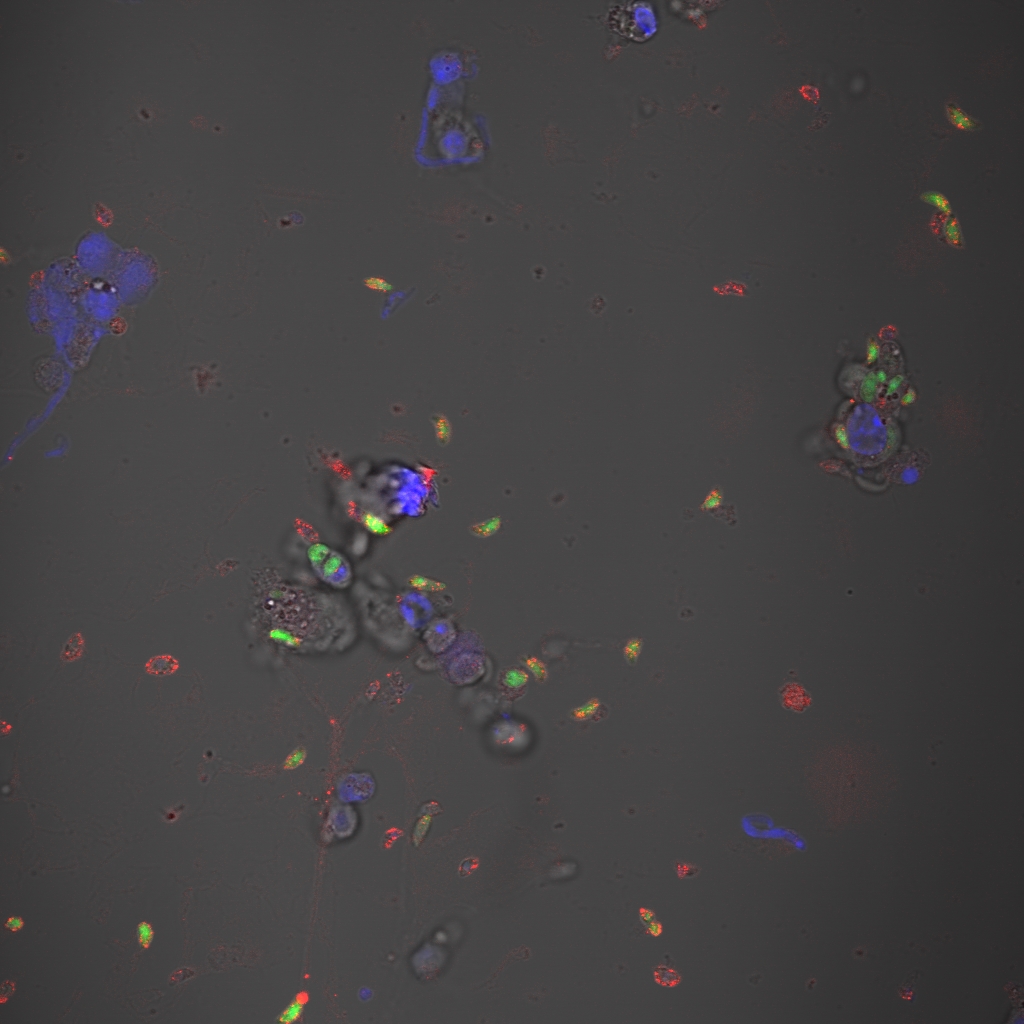

Supplement: Supplementary file 2 [file Data_Sheet_2.zip › Fig2/Fig2D/C3-LC3-5h/C3-LC3-5h.jpg]

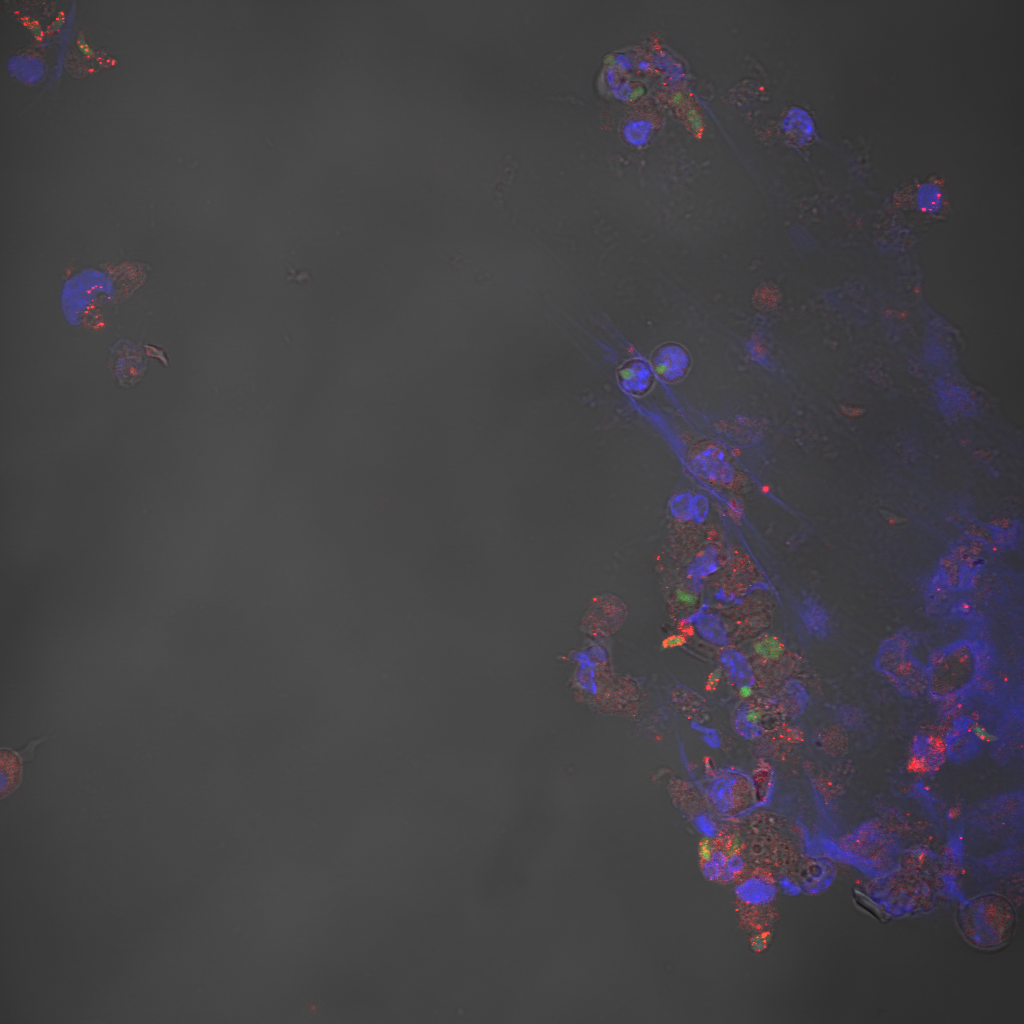

Supplement: Supplementary file 2 [file Data_Sheet_2.zip › Fig2/Fig2D/C3-LC3-7h/C3-LC3-7h.tiff]

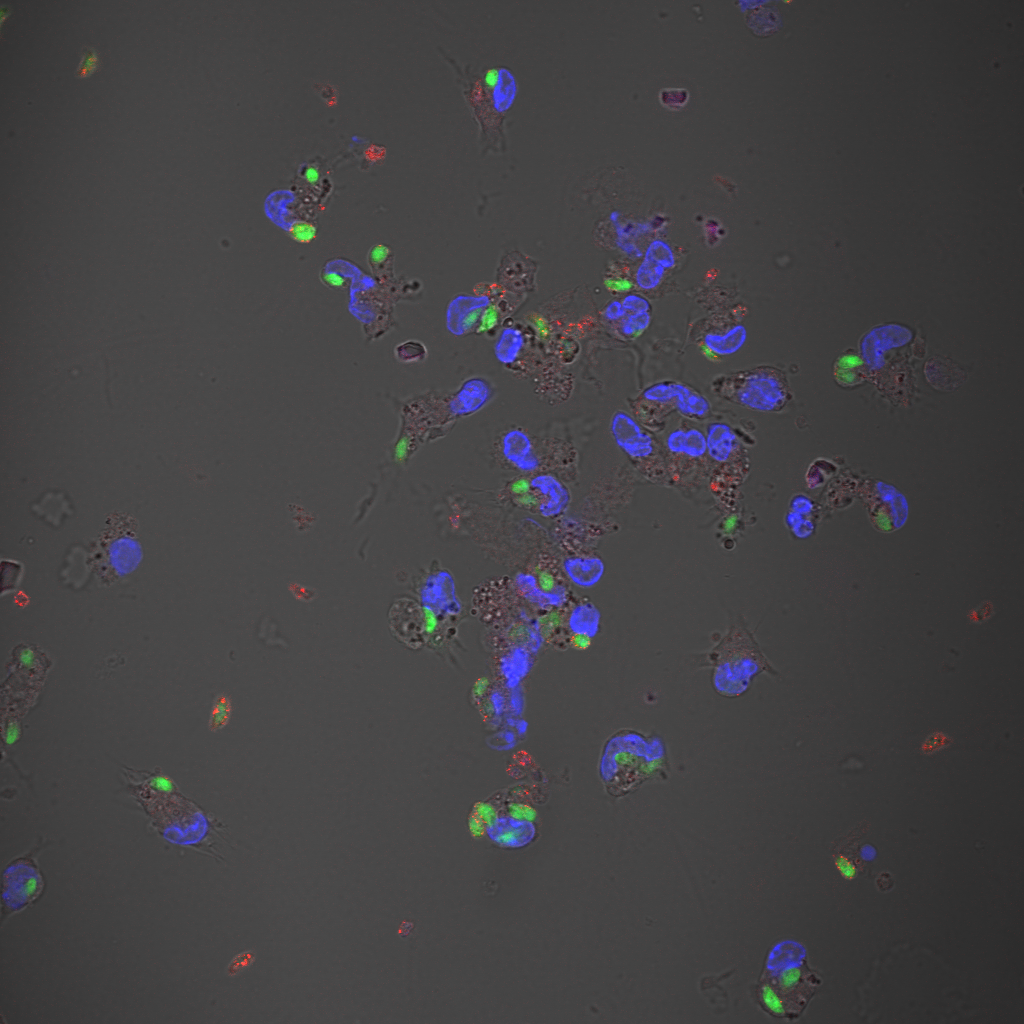

Supplement: Supplementary file 2 [file Data_Sheet_2.zip › Fig2/Fig2D/C3-LC3-9h/C3-LC3-9h.tif]

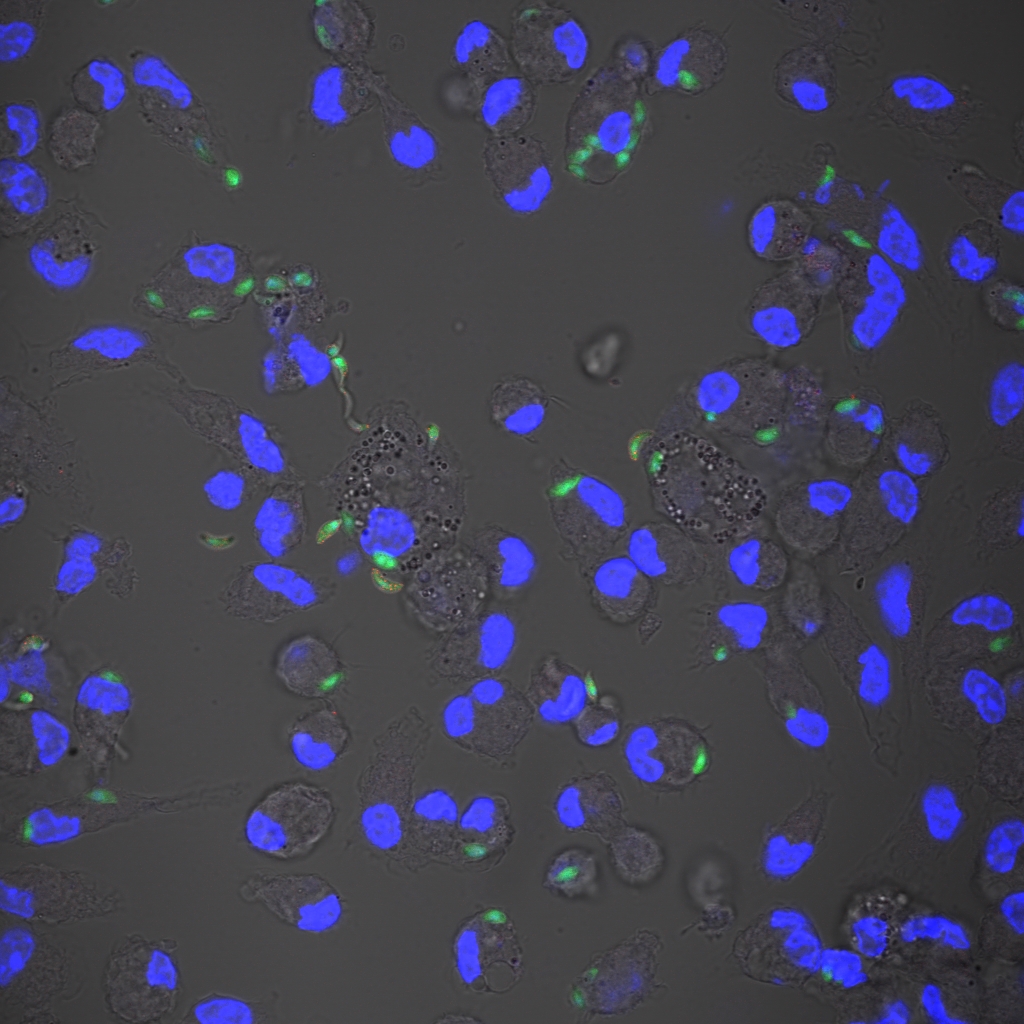

Supplement: Supplementary file 2 [file Data_Sheet_2.zip › Fig2/Fig2D/WT-LC3-1h/WT-LC3-1h.jpg]

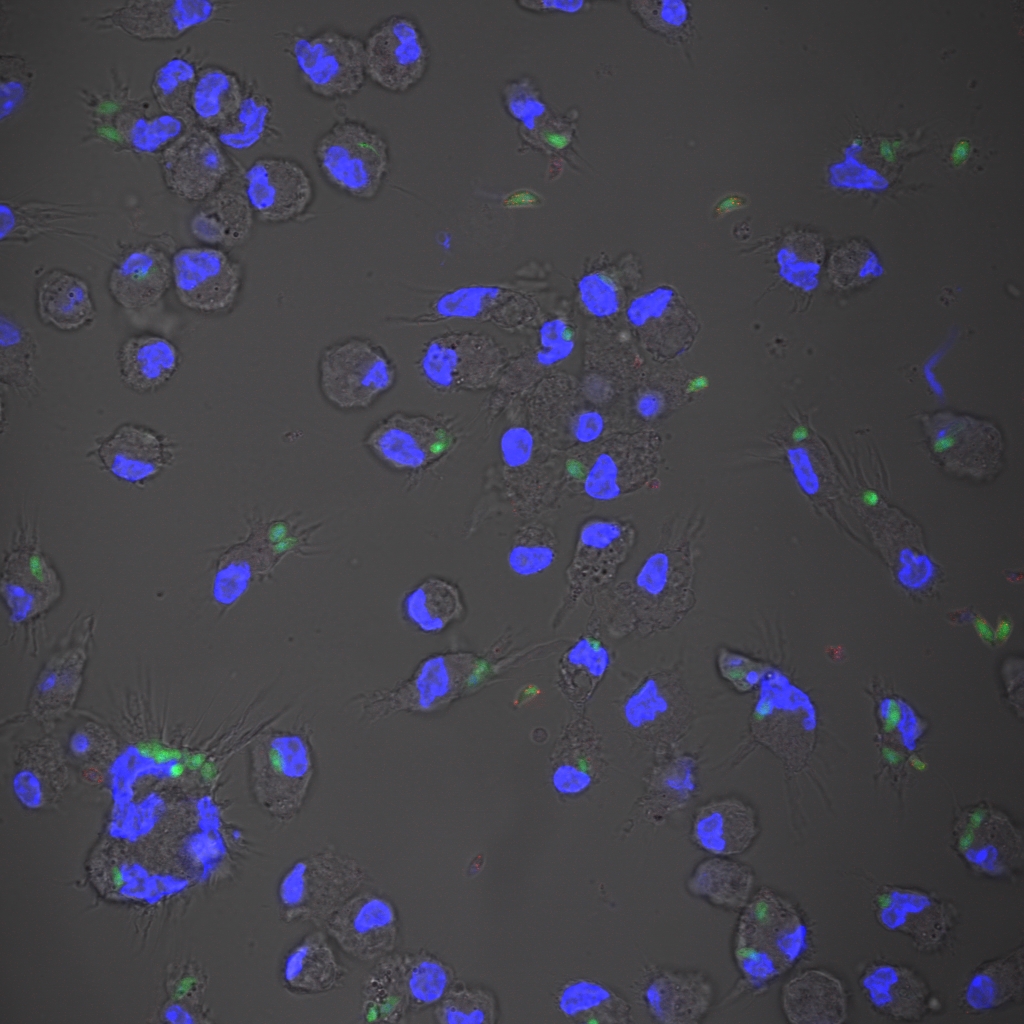

Supplement: Supplementary file 2 [file Data_Sheet_2.zip › Fig2/Fig2D/WT-LC3-3h/WT-LC3-3h.jpg]

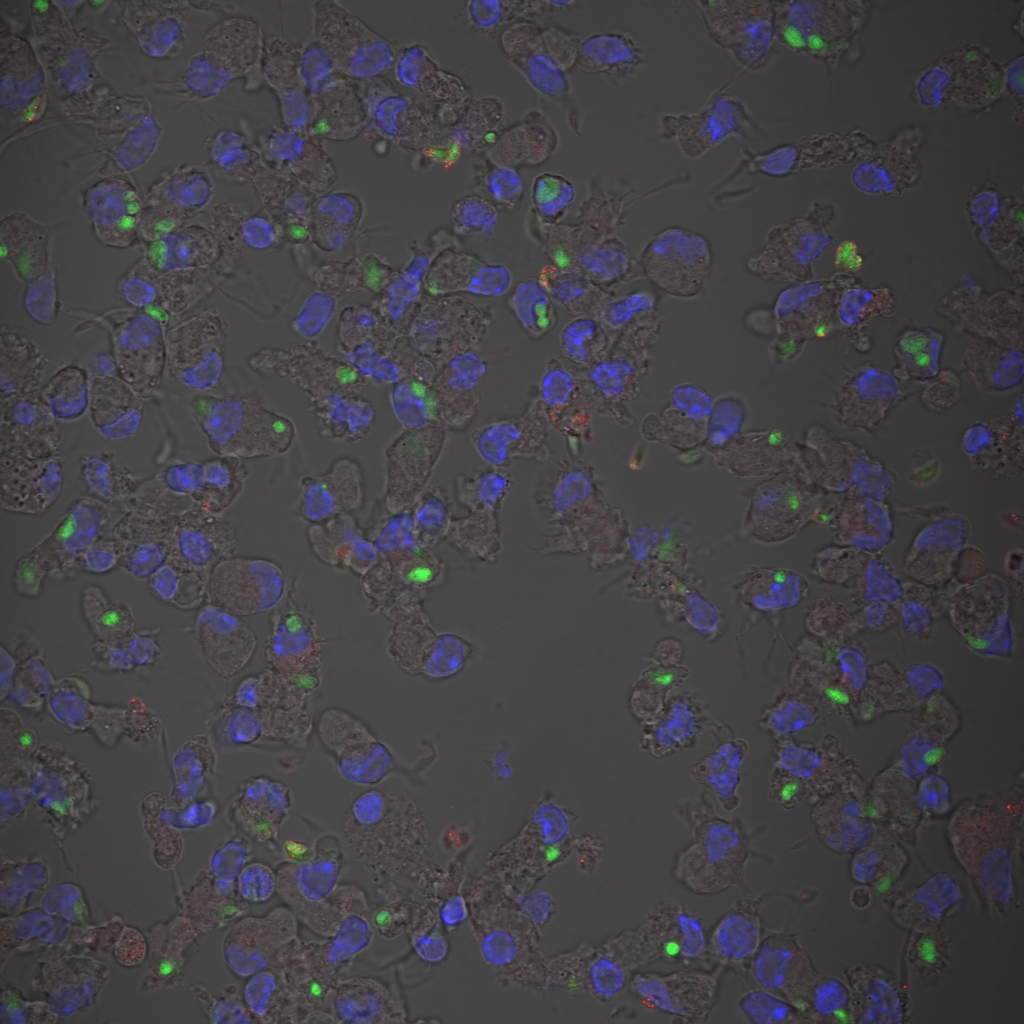

Supplement: Supplementary file 2 [file Data_Sheet_2.zip › Fig2/Fig2D/WT-LC3-5h/WT-LC3-5h.jpg]

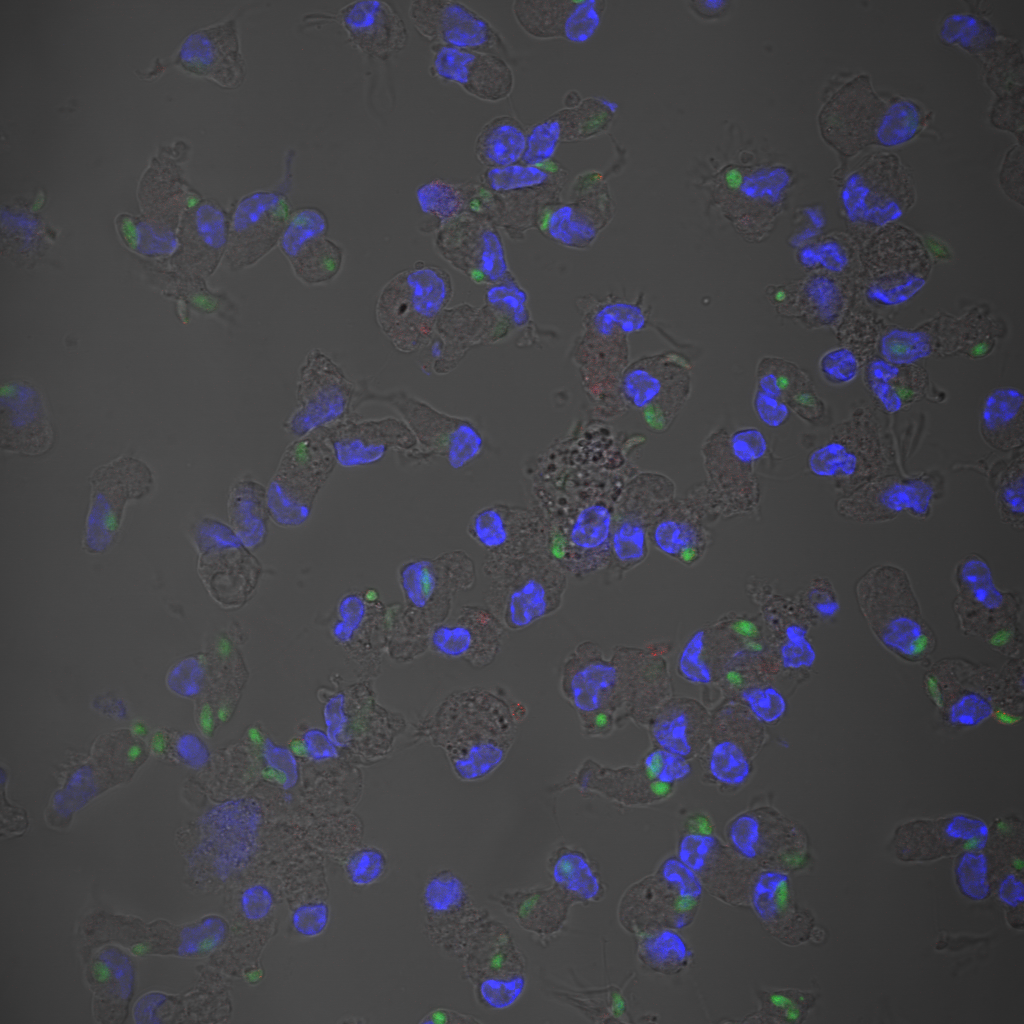

Supplement: Supplementary file 2 [file Data_Sheet_2.zip › Fig2/Fig2D/WT-LC3-7h/WT-LC3-7h.tif]

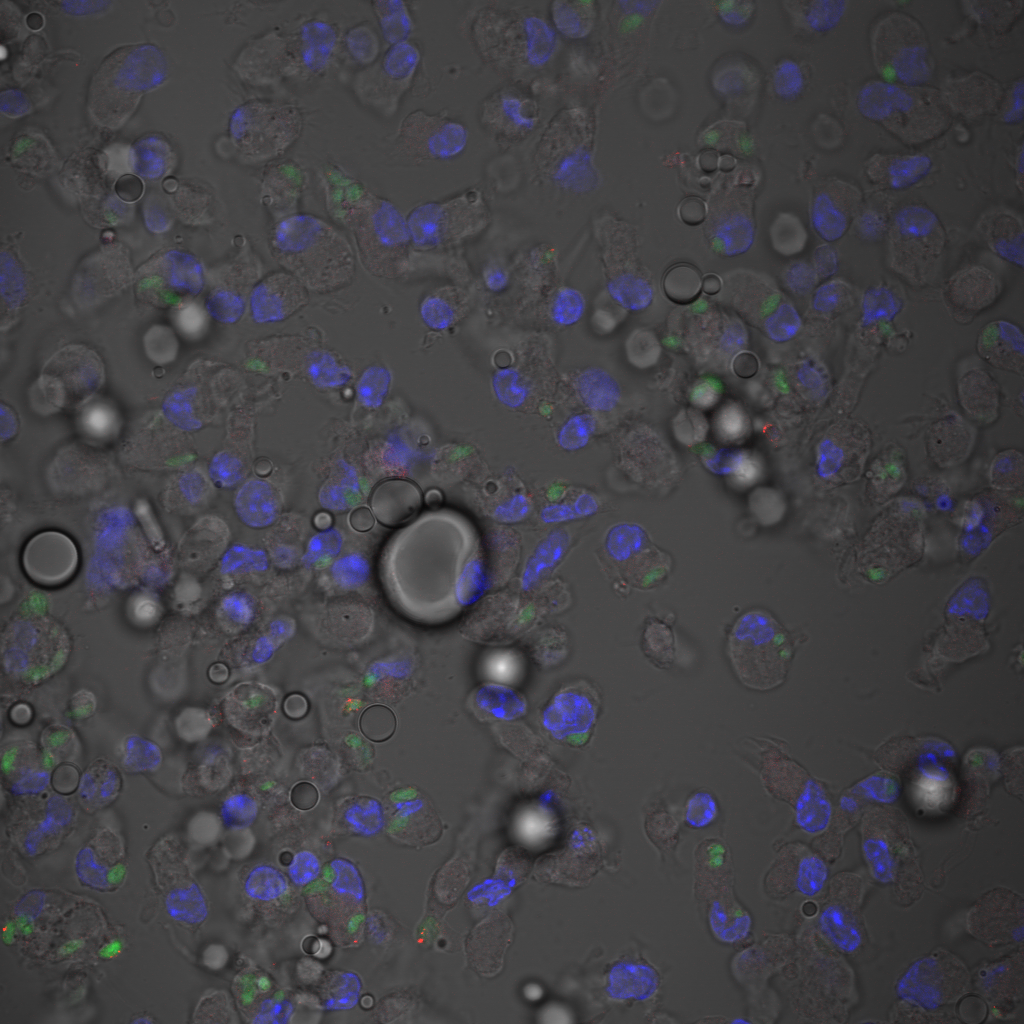

Supplement: Supplementary file 2 [file Data_Sheet_2.zip › Fig2/Fig2D/WT-LC3-9h/WT-LC3-9h.tif]

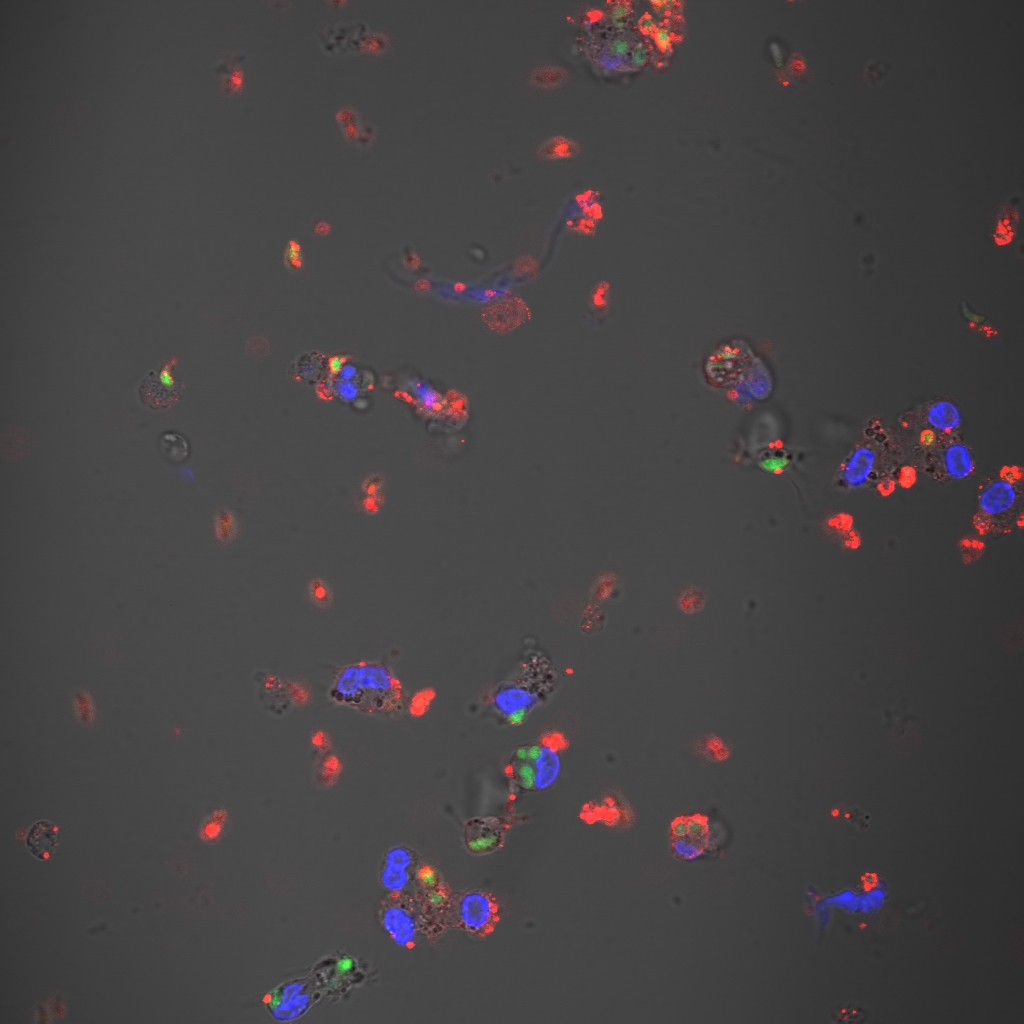

Supplement: Supplementary file 2 [file Data_Sheet_2.zip › Fig2/Fig2E/C3-LAMP-1-10h/C3-LAMP-1-10h.jpg]

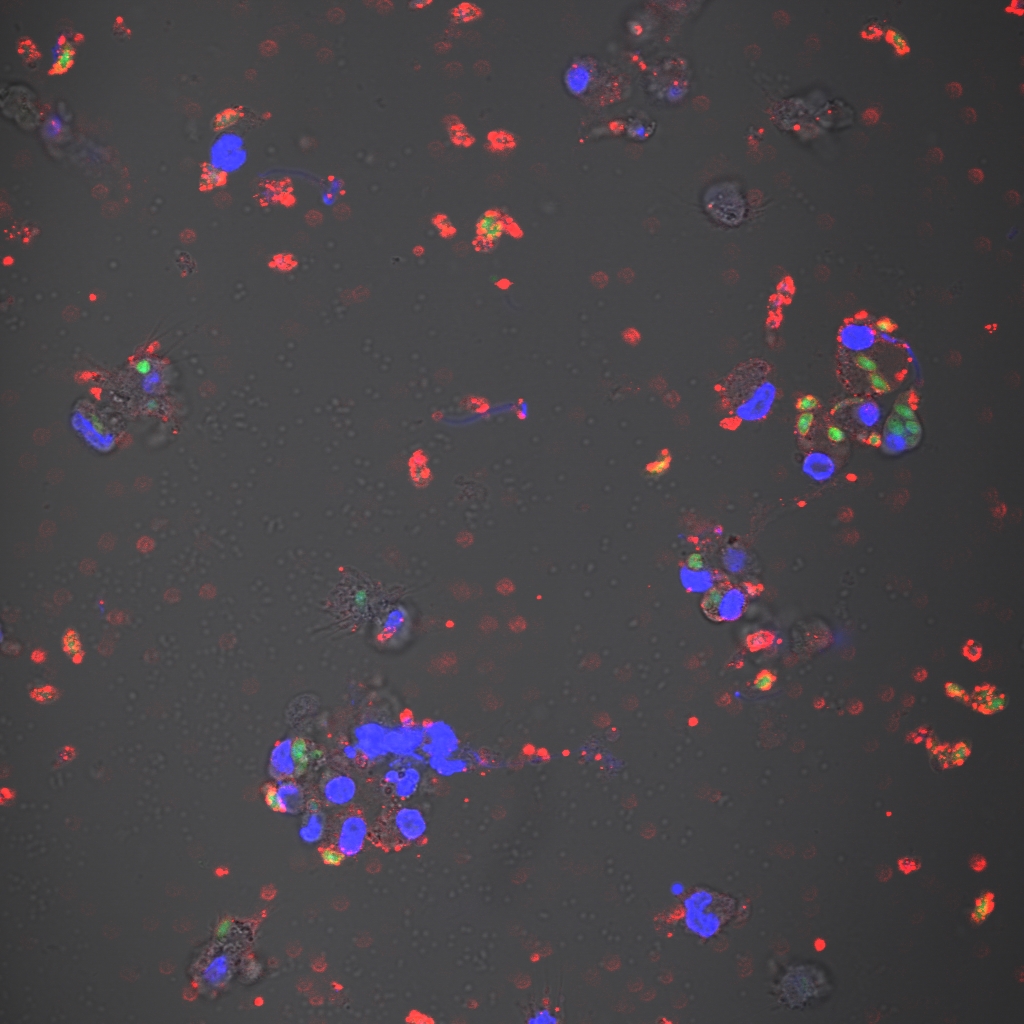

Supplement: Supplementary file 2 [file Data_Sheet_2.zip › Fig2/Fig2E/C3-LAMP-1-12h/C3-LAMP-1-12h.jpg]

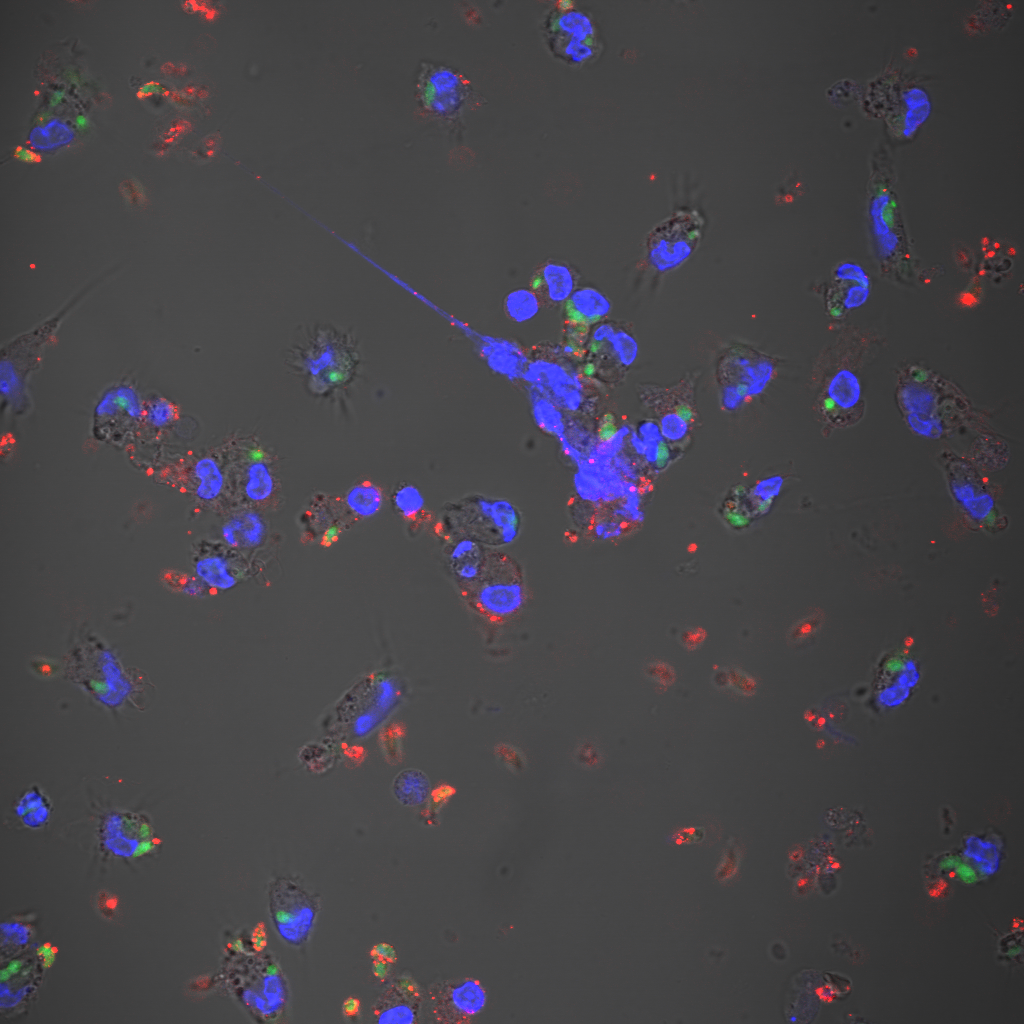

Supplement: Supplementary file 2 [file Data_Sheet_2.zip › Fig2/Fig2E/C3-LAMP-1-4h/C3-LAMP-1-4h.tiff]

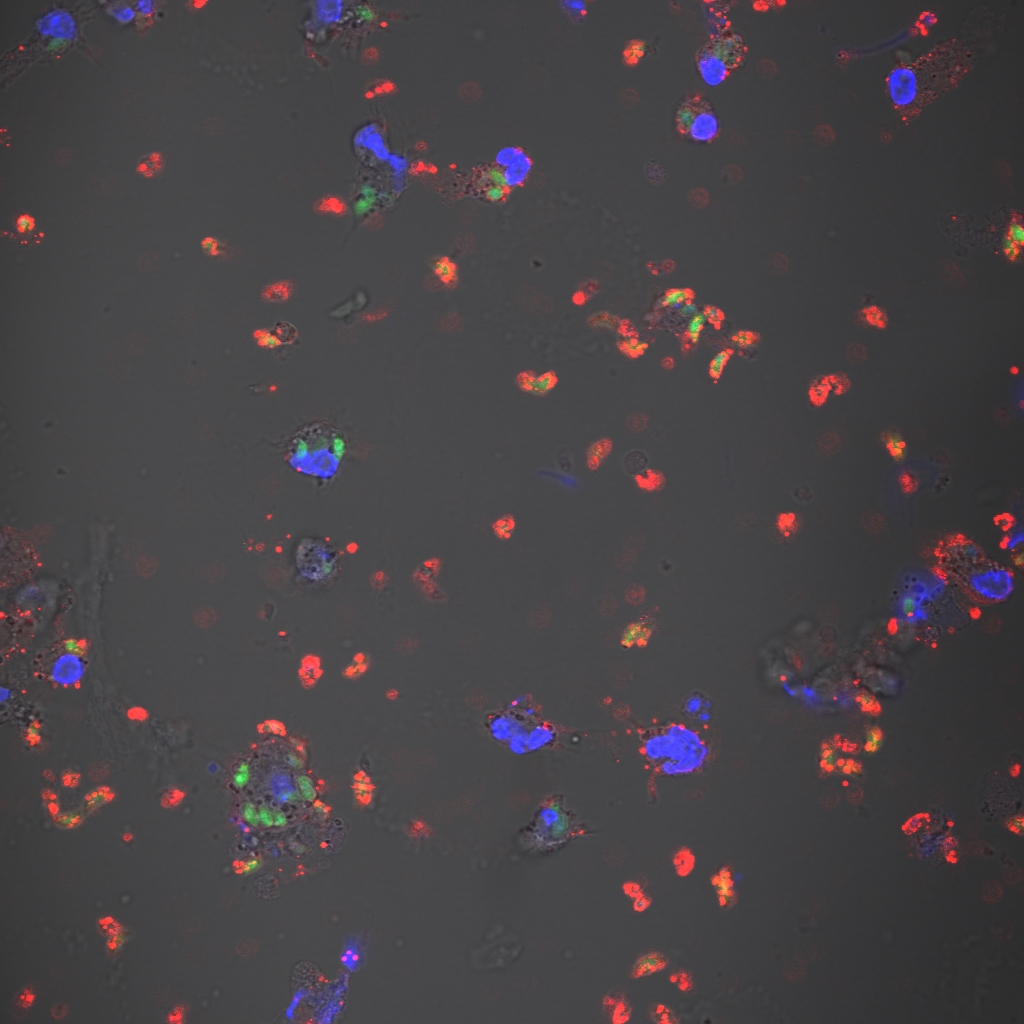

Supplement: Supplementary file 2 [file Data_Sheet_2.zip › Fig2/Fig2E/C3-LAMP-1-6h/C3-LAMP-1-6h.jpg]

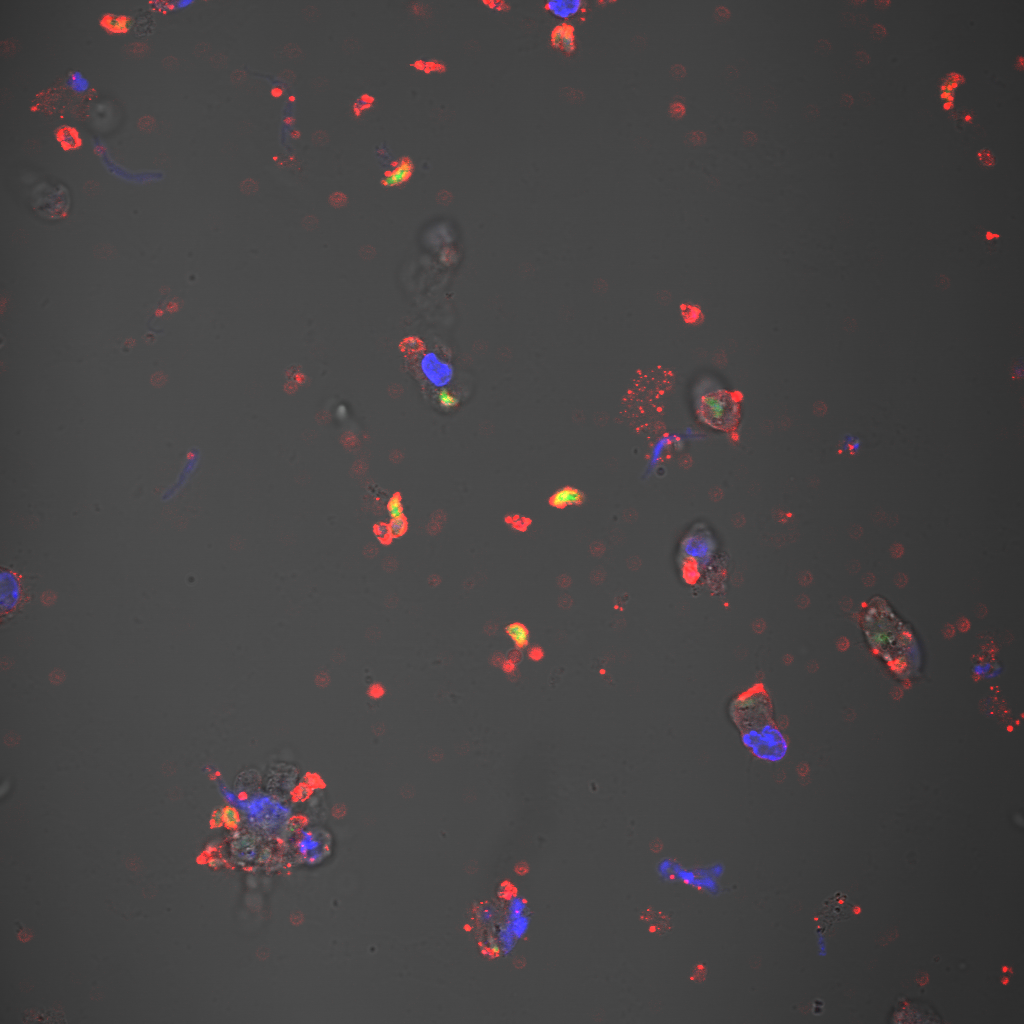

Supplement: Supplementary file 2 [file Data_Sheet_2.zip › Fig2/Fig2E/C3-LAMP-1-8h/C3-LAMP-1-8h.tif]

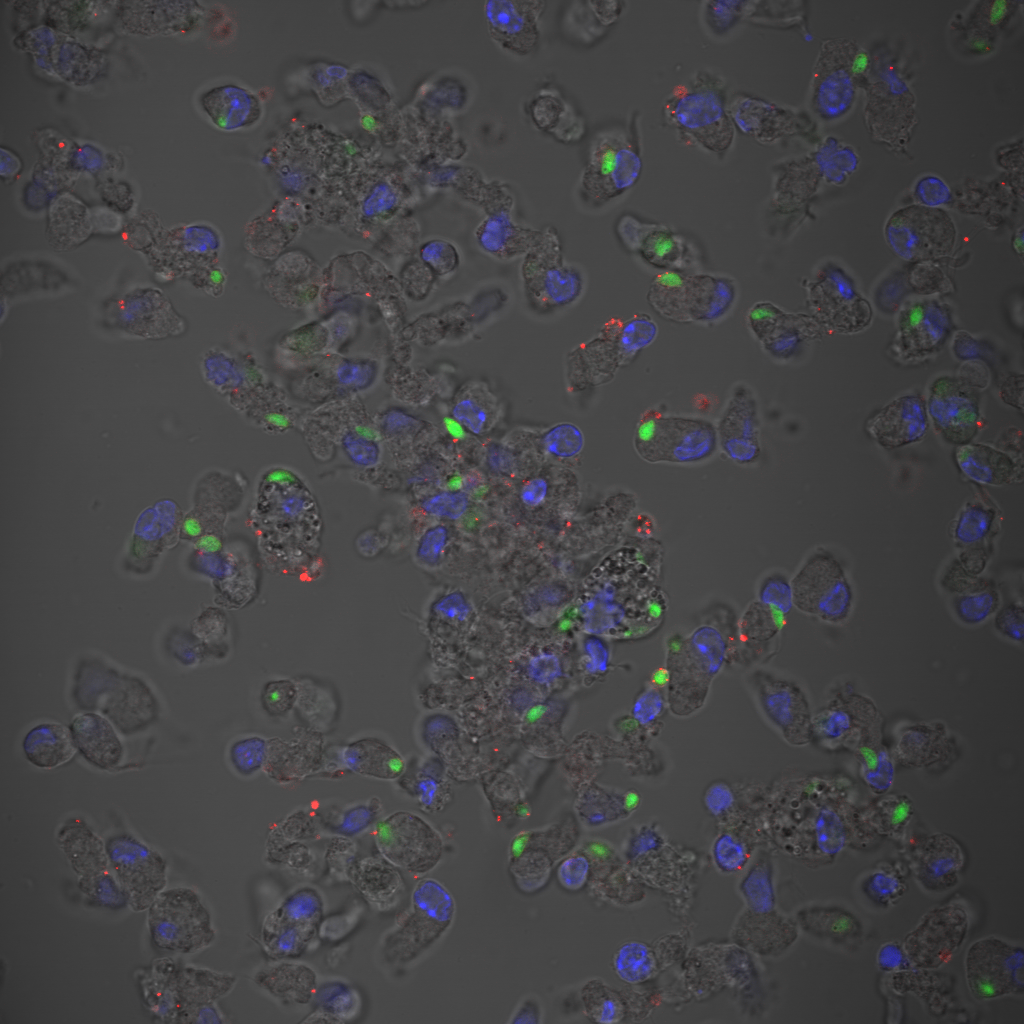

Supplement: Supplementary file 2 [file Data_Sheet_2.zip › Fig2/Fig2E/WT-LAMP-1-10h/WT-LAMP-1-10h.tif]

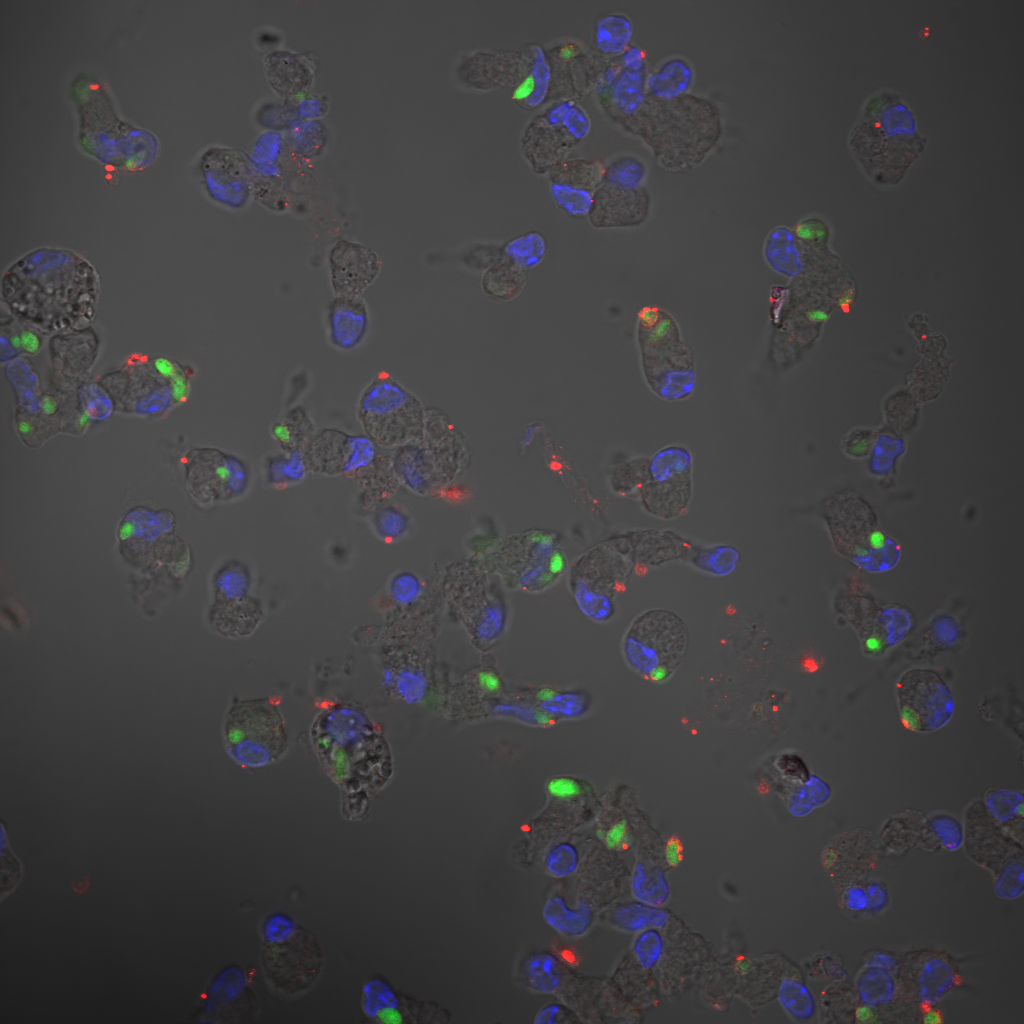

Supplement: Supplementary file 2 [file Data_Sheet_2.zip › Fig2/Fig2E/WT-LAMP-1-12h/WT-LAMP-1-12h.tif]

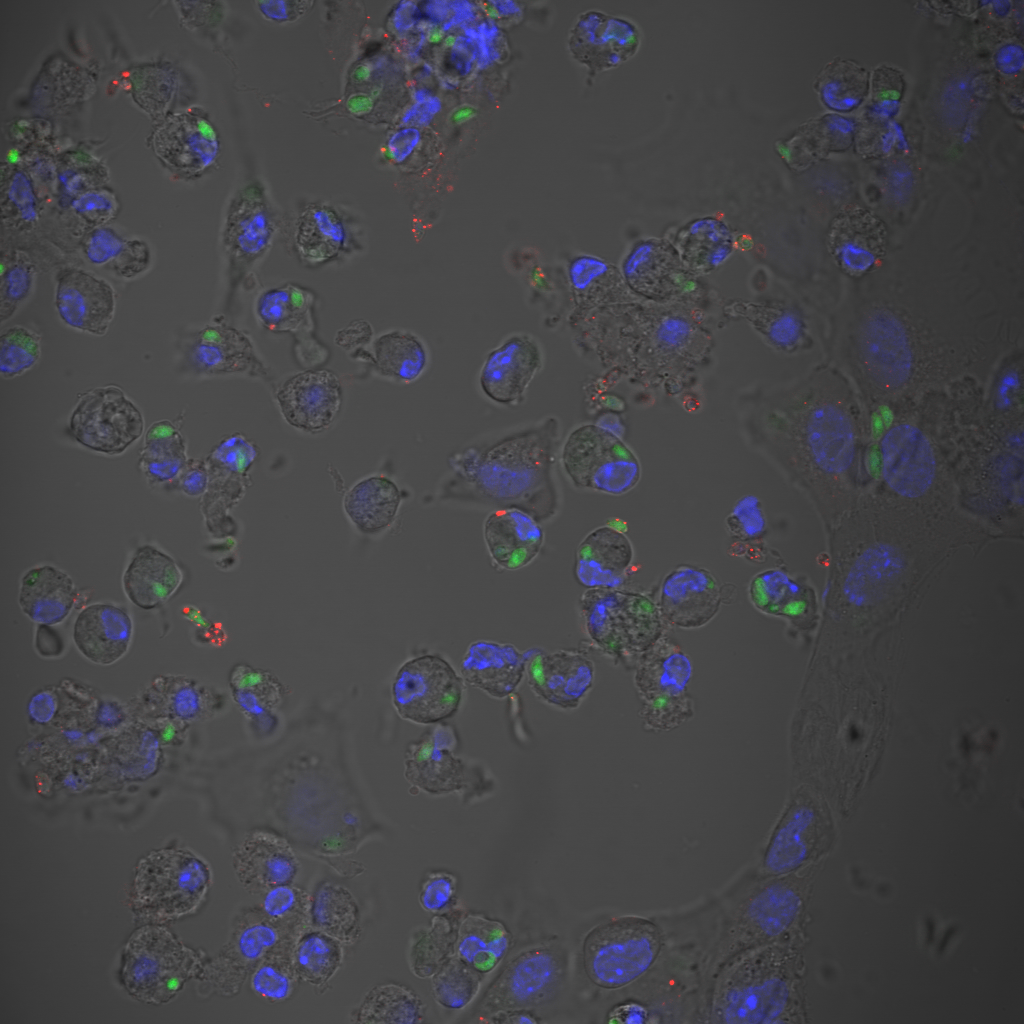

Supplement: Supplementary file 2 [file Data_Sheet_2.zip › Fig2/Fig2E/WT-LAMP-1-4h/WT-LAMP-1-4h.tif]

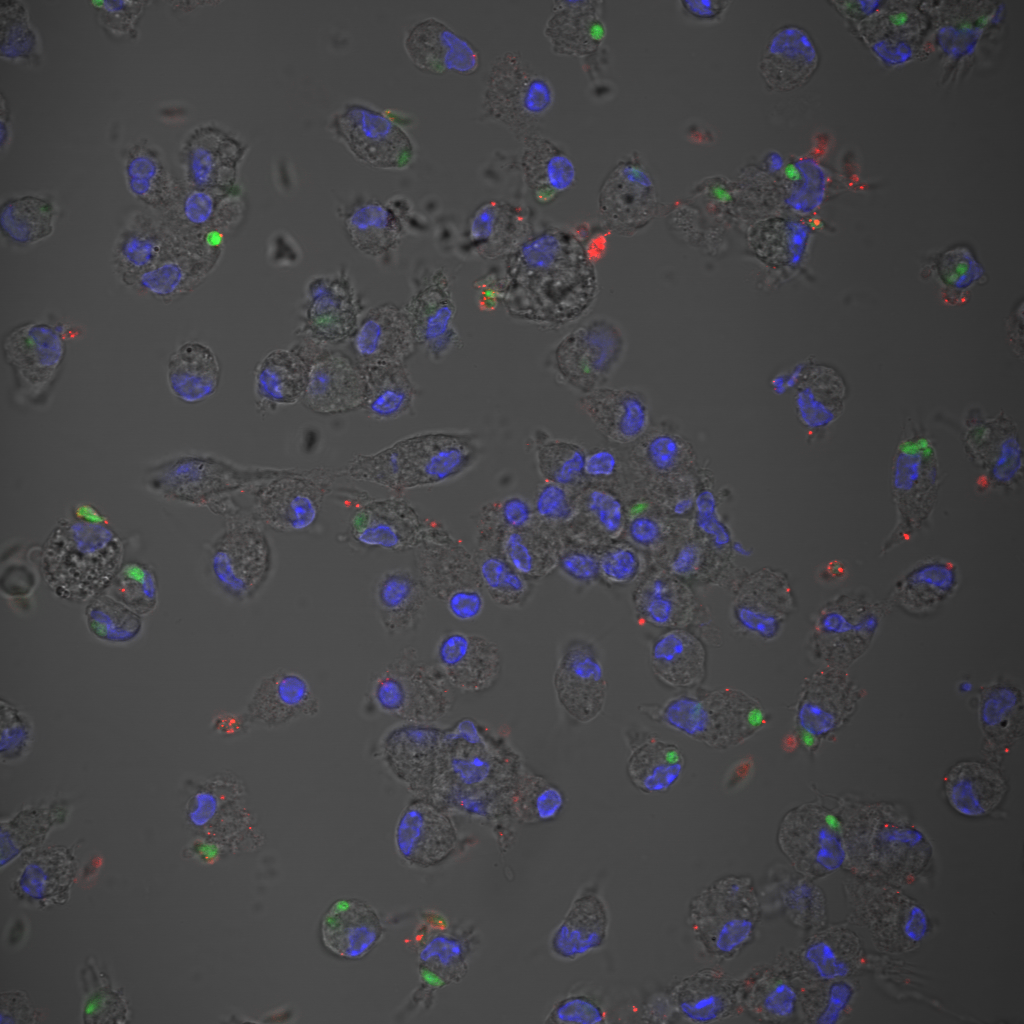

Supplement: Supplementary file 2 [file Data_Sheet_2.zip › Fig2/Fig2E/WT-LAMP-1-6h/WT-LAMP-1-6h.tif]

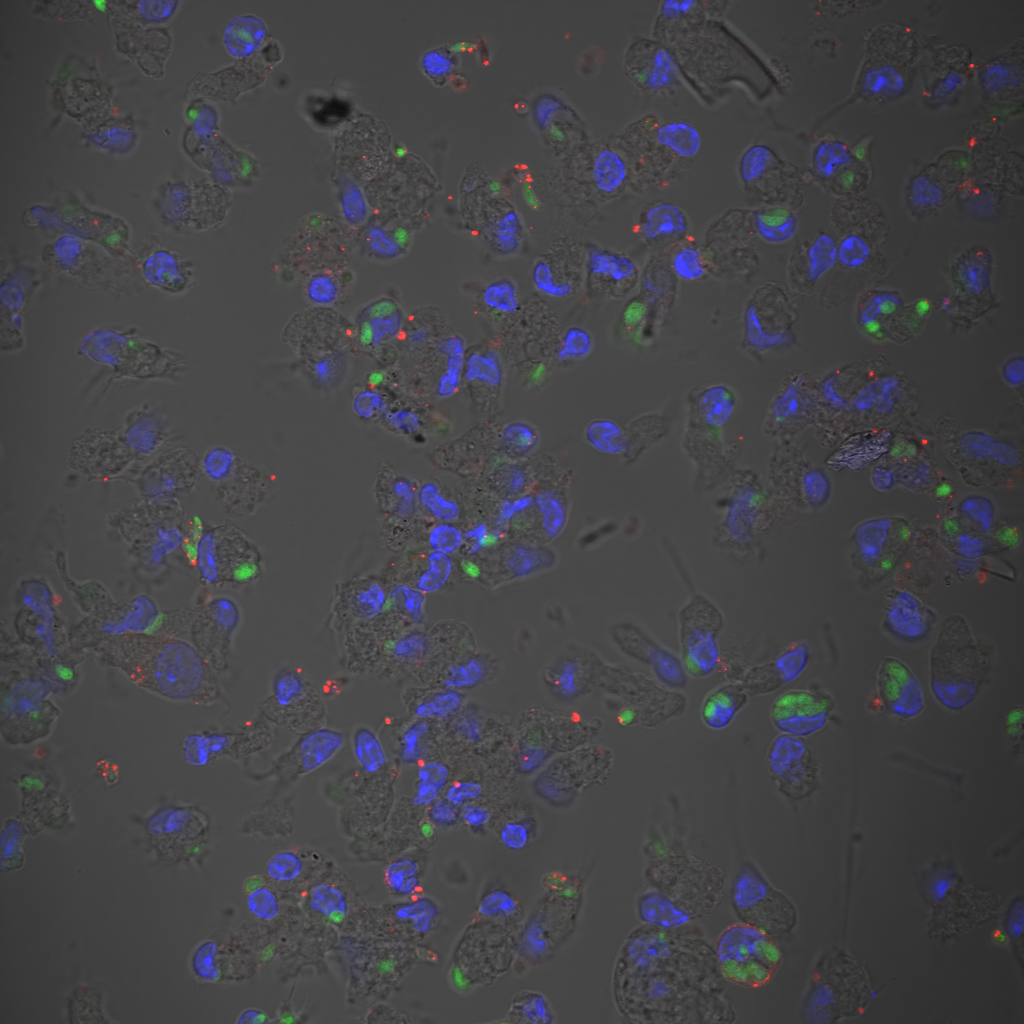

Supplement: Supplementary file 2 [file Data_Sheet_2.zip › Fig2/Fig2E/WT-LAMP-1-8h/WT-LAMP-1-8h.tif]

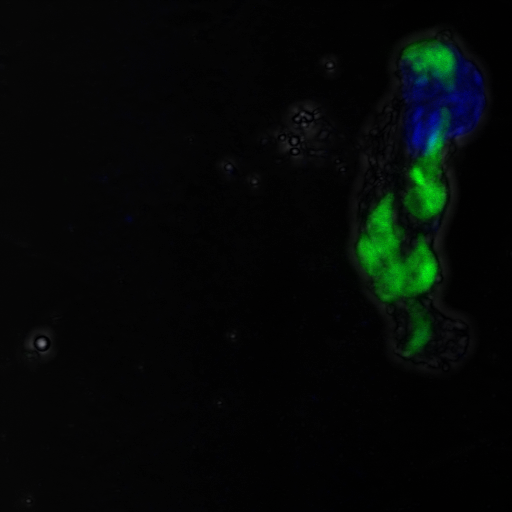

Supplement: Supplementary file 3 [file Data_Sheet_3.ZIP › Fig3/Fig3A/Fig2A-C3-Control.tif]

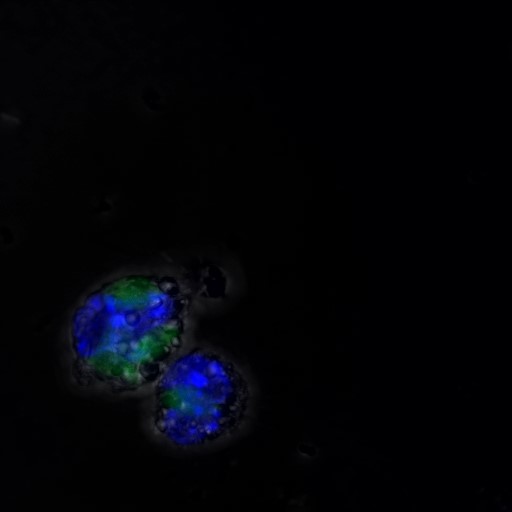

Supplement: Supplementary file 3 [file Data_Sheet_3.ZIP › Fig3/Fig3A/Fig3A-C3-IFN-a├.jpg]

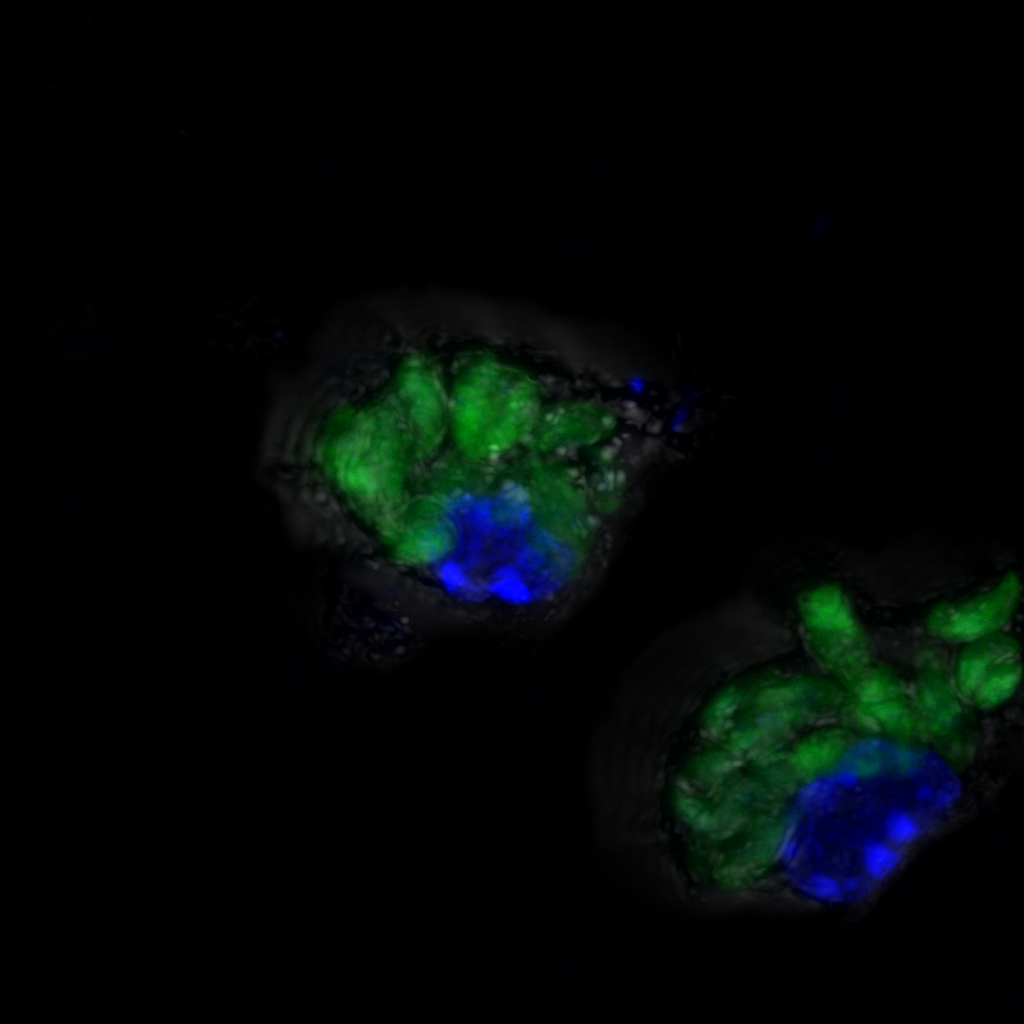

Supplement: Supplementary file 3 [file Data_Sheet_3.ZIP › Fig3/Fig3A/Fig3A-WT-Control.tif]

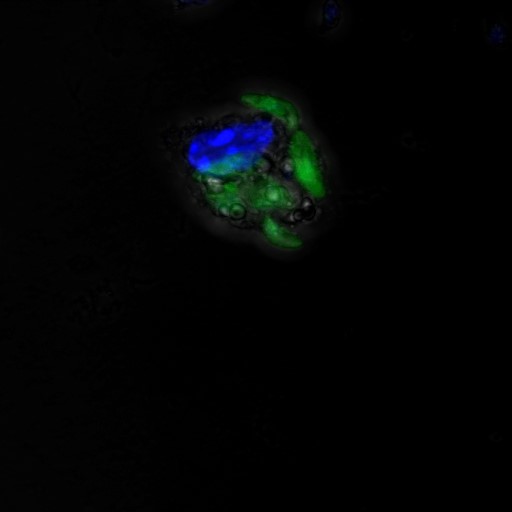

Supplement: Supplementary file 3 [file Data_Sheet_3.ZIP › Fig3/Fig3A/Fig3A-WT-IFN-a├.jpg]
